# When randomisation is not good enough: Matching groups in intervention studies

**DOI:** 10.3758/s13423-021-01970-5

**Published:** 2021-07-09

**Authors:** Francesco Sella, Gal Raz, Roi Cohen Kadosh

**Affiliations:** 1 Centre for Mathematical Cognition, Loughborough University, Loughborough, UK; 2 Department of Experimental Psychology, University of Oxford, Oxford, UK

**Keywords:** Variance minimisation, Randomised controlled trial, Research design, Clinical trials, Imbalance, Allocation methods

## Abstract

Randomised assignment of individuals to treatment and controls groups is often considered the gold standard to draw valid conclusions about the efficacy of an intervention. In practice, randomisation can lead to accidental differences due to chance. Researchers have offered alternatives to reduce such differences, but these methods are not used frequently due to the requirement of advanced statistical methods. Here, we recommend a simple assignment procedure based on variance minimisation (VM), which assigns incoming participants automatically to the condition that minimises differences between groups in relevant measures. As an example of its application in the research context, we simulated an intervention study whereby a researcher used the VM procedure on a covariate to assign participants to a control and intervention group rather than controlling for the covariate at the analysis stage. Among other features of the simulated study, such as effect size and sample size, we manipulated the correlation between the matching covariate and the outcome variable and the presence of imbalance between groups in the covariate. Our results highlighted the advantages of VM over prevalent random assignment procedure in terms of reducing the Type I error rate and providing accurate estimates of the effect of the group on the outcome variable. The VM procedure is valuable in situations whereby the intervention to an individual begins before the recruitment of the entire sample size is completed. We provide an Excel spreadsheet, as well as scripts in R, MATLAB, and Python to ease and foster the implementation of the VM procedure.

**Supplementary Information:**

The online version contains supplementary material available at 10.3758/s13423-021-01970-5.

## Introduction

### Randomisation in controlled trials

A common problem in intervention studies is comparing the effect of intervention while minimising the influence of confounding factors. In the pre-treatment assessment, a researcher usually measures the characteristics that the treatment aims to modify (i.e., outcome measures) as well as other variables that can exert an influence on the treatment (i.e., covariates). Then, the researcher will randomly assign individuals to the treatment and the control condition. In the ideal scenario, the control condition matches the treatment condition except for that specific feature of the treatment that the researcher considers to be crucial for causing a change in the outcome measures (e.g., placebo vs the active molecule in pharmacological studies). If the treatment is effective, the treatment group should improve in the outcome measures compared to the control group.

In the case of randomisation with large sample size, the statistical test for a difference at baseline or in other covariates becomes irrelevant as occurring significant differences reflect Type I error (de Boer et al., 2015; Roberts & Torgerson, 1999), which more likely arises when several covariates are considered (Austin et al., 2010). However, large sample sizes are difficult to achieve. Many researchers, especially in the clinical sciences, rely on small naturally occurring samples composed of individuals who voluntarily join the study when they wish to. In this scenario, the sampling is suboptimal as participants are not randomly sampled from the population, but they take part in the study based on convenience and opportunity. Although the assignment to different treatment conditions can be random, differences at baseline are more likely to emerge in small compared to large trials (Bruhn & Mckenzie, 2009; Chia, 2000; Nguyen & Collins, 2017; Saint-mont, 2015). Unfortunately, there is no statistical way to control for these differences between groups at pre-test (Miller & Chapman, 2001; Van Breukelen, 2006). Therefore, the imbalance in the pre-treatment scores can compromise the evaluation of the treatment efficacy, and seriously harm the interpretability of the results. To correct for this, the researcher may choose to allocate individuals to a condition based on previously collected pre-treatment scores and match the groups on these scores. However, this procedure requires the researcher to complete the pre-treatment assessment of all participants before the beginning of the treatment. The whole process may take several months, increase the attrition rate before the treatment begins and cannot account for unwanted changes in the measures of interest. Furthermore, the immediate implementation of the treatment is frequently necessary, especially in a clinical setting, where the treatment must begin in a critical phase of the patients’ clinical condition.

### Minimising group differences

One solution is the use of covariate-adaptive randomisation procedures (Chen & Lee, 2011; Dragalin et al., 2003; Endo et al., 2006; Scott et al., 2002), which allocate participants to the different conditions as they join the study and, at the same time, reduce the difference between groups on predefined critical variables. There are three commonly used types of covariate-adaptive randomisation methods: stratified randomisation, dynamic hierarchical randomisation, and minimisation (Lin et al., 2015). Differences at baseline can be reduced by using stratified randomisation, whereby specific (prognostic) variables are divided into strata and participants are randomly selected from each stratum. However, stratified randomisation becomes difficult to implement as the factors to control for increase (Therneau, 1993). In dynamic hierarchical randomisation, covariates are ranked in order of importance and participants are assigned to conditions via biased coin allocation when thresholds of imbalance are exceeded in selected covariates (Signorini et al., 1993). A minimisation procedure, the focus of this paper, calculates the level of imbalance in covariates that assigning a participant to each condition would cause, then allocates with high probability (to maintain a degree of randomness) the current participant to the condition that minimises the imbalance.

In this vein, the use of covariate-adaptive randomisation procedures not only matches groups on covariates, but also implicitly forces researchers to state in advance those critical covariates related to the treatment rather than controlling for their effect at a later stage, when running statistical analyses (Simmons et al., 2011). A covariate-adaptive randomisation procedure attempts to reduce the unwanted differences at baseline that inadvertently emerge from a random assignment. However, it is worth highlighting that the covariate-adaptive randomisation procedures aim to solve the imbalances at pre-test that might emerge from the random assignment of participants, rather than issues related to non-random selection of participants from naturally occurring samples.

Despite a variety of covariate-adaptive randomisation procedures at disposal, researchers conducting training/treatment studies, including randomised control trials (RCTs), seldom implement these methods (Ciolino et al., 2019; Lin et al., 2015; Taves, 2010). The lack of popularity of these procedures might be due to multiple factors. Researchers may feel more comfortable in implementing more traditional and easier to understand stratified/block randomisation. In this vein, an efficient implementation of covariate-adaptive procedures would require the consultancy of an expert statistician for the entire duration of the trial; an extra cost that principal investigators may prefer to avoid (Ciolino et al., 2019). Finally, the lack of free, easy-to-use, computerised functions to automatically implement covariate-adaptive procedures may have contributed to their still limited dissemination (Treasure & Farewell, 2012; Treasure & MacRae, 1998).

Here, we provide a procedure based on variance minimisation (VM; Frane, 1998; Pocock & Simon, 1975; Scott et al., 2002; Treasure & MacRae, 1998), which assigns the next incoming participant to the condition that minimises differences between groups in the chosen measures. Our procedure brings the benefit of using multiple covariates without creating strata in advance, as done in the stratified randomisation, and it is relatively easy to implement compared with the more complex dynamic hierarchical randomisation. The logic and the calculation behind the procedure are simple and easy-to-grasp also from an audience of non-experts. We provided ready-to-use code to implement the procedure using different (also free) software along with step-by-step written instructions, thereby reducing any costs associated with product licenses or consultancy from expert statisticians.

## Methods

### Description of the VM procedure

The goal of the VM procedure is to find the best group assignment for participants prior to an intervention, such that the groups are matched in terms of the scores that the researcher suspects might cause random differences in post-intervention outcomes. The VM procedure requires the researcher to define the number of groups to which participants can be assigned and to collect individual scores for each variable on which groups are matched. These variables can be continuous or binary, where nominal variables with more than two categories can be transformed into multiple dummy variables (as in regression analysis) before being passed to the VM procedure (see section Using VM Procedure on Non-Dichotomous Nominal Variables, in the Supplementary Materials). The procedure particularly suits those studies in which proper matching is essential, but the assignment to groups needs to occur while the recruitment is still ongoing. It works as follows.

**Fig. 1:**
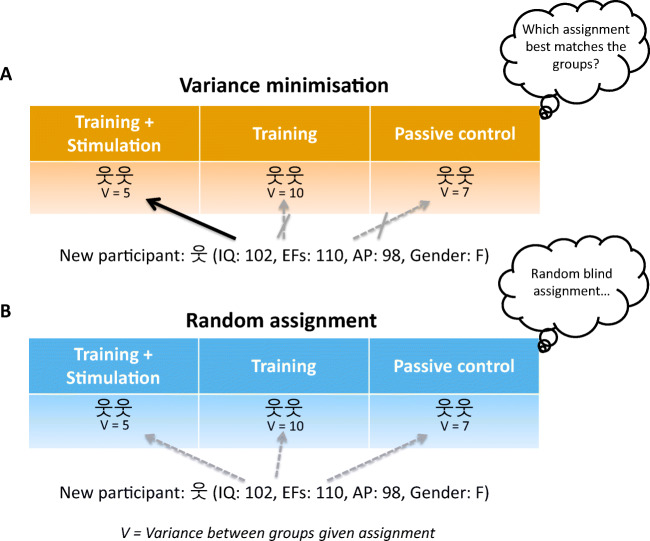
Comparison of assignment to groups using (**a**) variance minimisation and (**b**) random assignment. When a new participant joins a study, variance minimisation assigns the participant to the group that minimises the variance between groups along with the pre-defined variables (i.e., *V*); in this case intelligence (IQ), executive functions (EFs), attentional performance (AP), and gender, while keeping the number of participants in each group balanced. Random assignment, on the other hand, assigns the participant to every group with equal probability and does not match the groups

The first participants joining the study are sequentially assigned one to each group. For example, in case of three different groups (i.e., A, B, C), the first participant is assigned to Group A, the second participant to Group B, and the third participant to Group C. Then the fourth participant is added temporarily to each group, and for each temporary group assignment, the algorithm checks which group assignment for this participant would minimize the between-group variance (i.e., *V* in Fig. 1) of the measures of interest and assigns the participant to that group. The next (fifth) participant undergoes the same procedure, but the algorithm will not assign the present participant to the group of the previous participant in order to ensure a balanced distribution of participants in each condition. The same procedure goes on until there is only one group remaining, which in the case of three groups would be for the sixth participant. The sixth participant would be automatically assigned to the remaining group, such that each group would now have two participants assigned to them. Then, the entire procedure starts again with the possibility for the next participant to be assigned to all available groups (for a formal description of the variance minimisation procedure, see section Details of the Minimisation Procedure, in the Supplementary Materials).

To avoid predictable group assignments due to this shrinking set of available groups, the user can also specify a small probability of random assignment over the VM procedure (see section Discontinuous Implementation of the VM Procedure: The Parameter pRand, in the Supplementary Materials). This random component makes the assignment unpredictable even if the researcher has access to previous group allocations.

### Simulations

We present multiple simulations to illustrate how the VM procedure can be implemented in different scenarios and the advantages it provides.

In the first simulation, we implemented the VM procedure to assign participants to three experimental groups based on three continuous and one dichotomous variable. We compared the matching obtained from the VM procedure with random assignment. In the second simulation, we showed that the VM procedure better detects group differences and provides better estimates of effects compared with the attempt to *control for* the effect of covariates. In the supplementary materials, we demonstrate how to incorporate a random component in the VM procedure to ensure a non-deterministic assignment of participants to conditions (section Discontinuous Implementation of the VM Procedure: The Parameter pRand ) and how the VM can match participants also on non-dichotomous nominal variables (section Using VM Procedure on Non-Dichotomous Nominal Variables ). We briefly discuss the results of these two additional simulations in the Discussion section.

The functions to implement the VM procedure in Excel, MATLAB, Python, and R along with tutorials, as well as the R code of the simulation, can be found at the Open Science Framework (https://osf.io/6jfvk/?view_only=8d405f7b794d4e3bbff7e345e6ef4eed).

## Results

### VM procedure outperforms random assignment in matching groups on continuous and dichotomous variables

In the first fictional example, a researcher wants to evaluate whether the combination of cognitive training of executive functions and brain stimulation improves the clinical symptoms of ADHD. The study design comprises three groups: the first group receives brain stimulation and the executive functions training; the second group receives sham stimulation and the training; the third group receives neither training nor stimulation (passive control group). The researcher aims to match the three groups on intelligence, executive functions performance, attentional performance, and gender. Figure 1 illustrates how VM assigns incoming participants compared with a traditional random assignment.

We simulated 1,000 data sets whereby we randomly drew the scores for IQ, executive functions, and attentional performance from a normal distribution, with a mean of 100 and a standard deviation of 15. Participants’ gender came from a binomial distribution with the same probability for a participant to be male or female. The simulated values for the matching variables were randomly generated, therefore there were no real differences between groups. We varied the sample size to be very small (*n* = 36), small (*n* = 66), medium (*n* = 159), and large (*n* = 969), reflecting the researcher’s intention to evaluate the possible presence of an extremely large (*f* = 0.55), large (*f* = 0.40), medium (*f* = 0.25), and small (*f* = 0.10) effect size, respectively, while keeping the alpha at .05 and power at 80% (Faul et al., 2009). We assigned participants to the three groups randomly or by using the VM procedure.

**Fig. 2:**
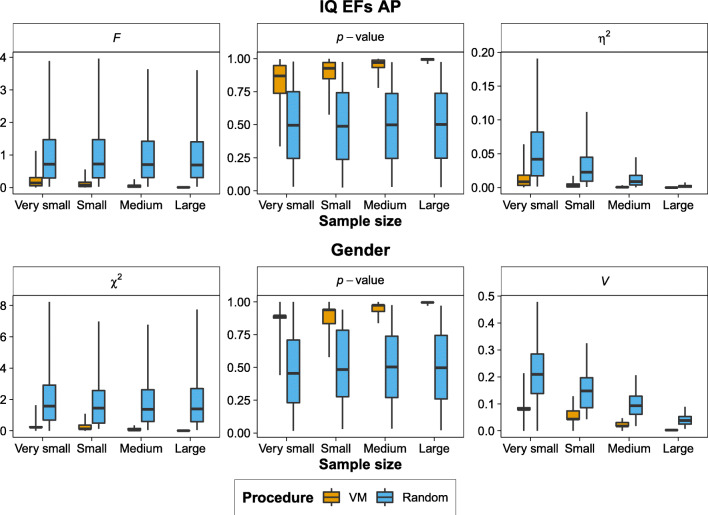
A comparison of the VM procedure and random assignment based on simulated data. Top panel: Distributions of *F*-values, *p*-values, and η^2^ values from ANOVAs comparing groups on intelligence (IQ), executive functions (EFs), and attentional performance (AP) separately for the VM procedure (orange boxplots) and the random assignment (blue boxplots). Bottom panel: Distributions of χ^2^, *p*-values, and Cramer’s *V* values comparing groups on gender separately for the VM procedure (orange boxplots) and the random assignment (blue boxplots). The boxplots represent the quartiles whereas the whiskers represent the 95% limits of the distribution. (Colour figure online)

We ran univariate analyses of variance (ANOVAs) with IQ, executive functions, and attentional performance as dependent variables and group as factor whereas differences in gender distribution across groups were analysed using χ^2^ tests. In Fig. 2, we show the distributions of *F*, *p*, and η^2^ values from ANOVAs on IQ, executive functions, and attentional performance (top panel), whereas in the case of gender, we presented the distribution and χ^2^, *p*, and Cramer’s *V* values (bottom panel) separately for the random assignment and the VM procedure across different sample sizes. Compared with random assignment, the VM procedure yielded smaller *F*, η^2^, χ^2^, and Cramer’s *V* values and the distribution of *p*-values was skewed toward 1, rather than uniform. The VM procedure demonstrated an efficient matching between groups starting from a very small sample size while keeping the number of participants in each group balanced. Moreover, both the VM procedure and the random assignment violated ANOVA assumptions on the normality of residuals and homogeneity of variance between groups with a similar rate (see Supplementary Materials, Fig. S1).

### Matching groups on a covariate versus controlling for a covariate with imbalance

We simulated an intervention study to display the advantages that the minimisation procedure provides in terms of detecting group differences and better estimates of effects compared with the attempt to *control for* the effect of covariates in the statistical analysis after the intervention was completed. A researcher evaluates the effect of an intervention on a dependent variable Y while controlling for the possible confounding effect of a covariate A, which positively correlates with Y, and a covariate B that correlates with covariate A (i.e., pattern correlation 1), or Y (i.e., pattern correlation 2), or neither of them (i.e., pattern correlation 3). In this vein, the covariate A represents a variable that the researchers ought to control for, given its known relation with the dependent variable Y, whereas the covariate B represents a non-matching variable that is still inserted into the model as it might have a real or spurious correlation with the covariate A and the dependent variable Y. We simulated a small, medium, and large effect of the intervention (i.e., Cohen’s *d* = 0.2; *d* = 0.5; *d* = 0.8) and, accordingly, we varied the total sample size to be 788, 128, and 52 to achieve a power of 80% while keeping the alpha at .05 (Faul et al., 2009). For comparison, we used the same sample sizes, 788, 128, and 52, when simulating the absence of an intervention effect (i.e., Cohen’s *d* = 0). Crucially, we compared the scenario whereby the researcher matches participants on the covariate A (i.e., VM on CovA) before implementing the intervention or randomly assigns participants to the control and training group and then attempts to control for the effect of covariate after the intervention (i.e., Control for CovA). The subsequent inclusion of the covariate A in the analysis, especially in the case of imbalance between groups in the covariate A, would bias the effect of the group on Y when the difference between groups in the covariate A is larger in the direction of the intervention effect. Conversely, the minimisation procedure reduces the difference between groups on the covariate A and the inclusion of the covariate A into the analysis (i.e., analysis of covariance; ANCOVA) would not cause biases in the estimation of the effect of the group on Y.

In the case of the control for covariate approach, we generated the scores of the covariate A by taking them from a standard normal distribution (*M* = 0, *SD* = 1) and we randomly assigned participants to the control and training group. We generated an imbalance in the covariate A by calculating the standard error of the mean and multiplying it for the standard normal deviates ±1.28, ±1.64, ±1.96 corresponding to the 20%, 10%, and 5% probabilities respectively of the standard normal distribution. The use of the standard error allowed to keep the imbalance proportionate to the sample size. The obtained imbalance was added to the scores of the covariate A only for the training group, thereby generating a difference in covariate A that went in the same or in the opposite direction with respect to the intervention effect (i.e., larger scores on the dependent variable only for the training group; Egbewale et al., 2014). We also included the case of absent imbalance for reference. In the case of the VM procedure, we took the previously generated scores of the covariate A with the imbalance, and we assigned participants to the control or training group using the VM procedure. Then, we generated the scores of Y that were correlated with the covariate A according to four correlations, that were, 0, 0.5, 0.7, and 0.9. Finally, we added 0, 0.2, 0.5, 0.8 to the Y scores of the training group to simulate an absent, small, medium, and large effect of the intervention. 

In both the random assignment and the VM procedure, the covariate B was generated to alternatively have a correlation of 0.5 (*SD* = 0.1) with the covariate A (i.e., Pattern 1), Y (i.e., Pattern 2), or no correlation with these two variables (i.e., Pattern 3). We randomly selected the correlation from a normal distribution with an average 0.5 and standard deviation of 0.1 to add some noise to the correlation while maintaining it positive and centred on 0.5.

Overall, we varied multiple experimental conditions in 504 scenarios (for a similar approach, see Egbewale et al., 2014):

- seven imbalances on the covariate A: −1.96, −1.64, −1.28, 0, 1.28, 1.64, 1.96;
- four correlations between covariates A and Y: 0, 0.5, 0.7, 0.9;
- six treatment effects: 0 (×3 as the absence of the effect was tested with three sample sizes, that were, 52, 128, 788), 0.2, 0.5, 0.8;
- three patterns of correlation between the covariate B, covariate A, and Y.

We simulated each scenario 1,000 times.

As expected, the correlations between the covariate B and the other two variables varied according to the pre-specified patterns of correlations, which were practically identical in the VM and control for covariate approach (see Table S1 in the Supplementary Materials).

We ran a series of ANCOVAs with Y as the dependent variable, the covariates A and B, and group [Training, Control] as independent variables. We used a regression approach as the variable group was converted to a dichotomous numerical variable (i.e., control = 0, training = 1) to directly use the regression coefficients as estimates for the effect of each variable on Y. Both the VM procedure and the control for the covariate approach display a similar rate in violating ANCOVA assumptions of the normality of residuals and homogeneity of variance between groups (see Supplementary Materials; Fig. S2).

**Fig. 3:**
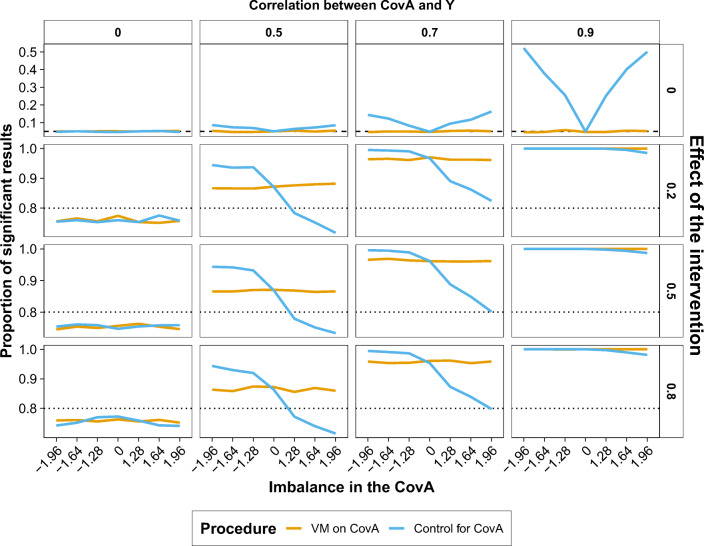
Proportion of significant results (*y*-axis) for the effect of group in the ANCOVA (Y ~ CovA + CovB + Group) separately for the VM procedure (orange lines) and control for CovA approach (blue lines) across imbalances of the covariate A (*x*-axis) when the sample size varied according to the effect size to be detected (rows; absent = 0, *n* = 788; small = 0.2, *n* = 788; medium = 0.5, *n* = 128; large = 0.8, *n* = 52) and the correlation between the covariate A and the dependent variable Y ranged between 0 and 0.9 (columns). The black dotted line represents alpha (i.e., 0.05) and the dashed black line represents the expected power (i.e., 0.8). (Colour figure online)

**Fig. 4:**
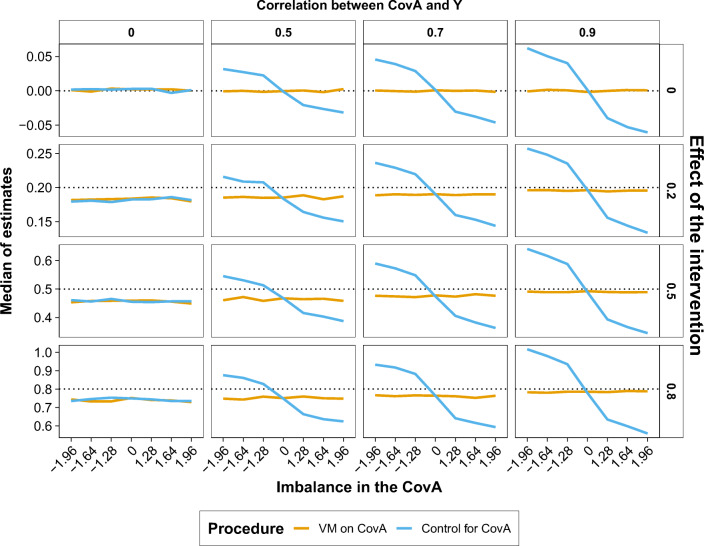
Median of estimates (*y*-axis; regression coefficients) for the effect of group in the ANCOVA (Y ~ CovA + CovB + Group) separately for the VM procedure (orange lines) and control for CovA approach (blue lines) across imbalances of the covariate A (*x*-axis) when the sample size varied according to the effect size to be detected (rows; absent = 0, *n* = 788; small = 0.2, *n* = 788; medium = 0.5, *n* = 128; large = 0.8, *n* = 52) and the correlation between the covariate A and the dependent variable Y ranged between 0 and 0.9 (columns). The black dotted line represents the expected regression coefficients (i.e., 0, 0.2, 0.5, 0.8). (Colour figure online)

In this fictitious scenario, the researcher would be interested in evaluating the effect of the group on Y while controlling for covariates. Therefore, we reported the proportion of significant results (*p* < .05; Fig. 3) and the estimated effect (i.e., coefficient of the regression; Fig. 4) for the effect of group on Y depending on the imbalance in the covariate A, the effect size of the intervention, and the degree of correlation between the covariate A and Y. For simplicity, in Figs. 3 and 4, we reported only the simulation with a large sample size (i.e., *n* = 788) when the effect of the intervention was absent (i.e., *d*=0). The pattern of results remained stable across the patterns of correlations of the covariate B. Therefore, we reported the proportion of significant results and estimated effects for the group, covariate A, and covariate B across the patterns correlation of the covariate B in the Supplementary Materials (Figs. S5–S22).

When the effect of the intervention was present (second to fourth rows in Fig. 3), the VM procedure showed a more stable detection of significant results also in the presence of serious imbalances in the covariate A. This stability became clearer as the correlation between the covariate A and Y increased. When the effect of the intervention was absent (first row in Fig. 3), the VM procedure always kept the Type I error around 0.05 while the control covariate approach inflated Type I error rate in the case of strong imbalance in the covariate A when it was highly correlated (i.e., 0.7, 0.9) with the outcome variable Y.

A similar pattern of results emerged when we compared the estimates of the effect of the group (i.e., regression coefficients) yielded by the VM procedure and the control for covariate approach. The VM procedure always provided accurate estimates of the effect of the group. Conversely, the control for covariate approach returned biased estimates with large imbalances in the covariate A and when its correlation with the outcome variable Y was high (i.e., 0.7, 0.9; Fig. 4).

## Discussion

In treatment studies, groups should be as similar as possible in all the variables of interest before the beginning of the treatment. An optimal matching can ensure that the effect of the treatment is not related to the pre-treatment characteristics of the groups and can, therefore, be extended to the general population. In contrast, the random assignment can yield relevant, and even statistically significant, differences between the groups before the treatment (Treasure & MacRae, 1998).

The proposed VM procedure constitutes a quick and useful tool to match groups before treatment on both continuous and categorical covariates (Pocock & Simon, 1975; Scott et al., 2002; Treasure & MacRae, 1998). The latter, though, need to be transformed into dummy variables to be passed to the minimisation algorithm (for a minimisation procedure that directly handles nominal covariates see Colavincenzo, 2013). We simulated an intervention study whereby a researcher used the VM procedure on a covariate to assign participants to a control and intervention group rather than controlling for the covariate at the analysis stage. Among other features of the simulated study, we manipulated the correlation between the matching covariate and the outcome variable and the presence of imbalance between groups in the covariate. Controlling for covariates post hoc inflated Type I error rate and yielded biased estimates of the effect of the group on the outcome variable when the imbalance between groups in the covariate increased and the correlation between the covariate and the outcome variable was high. Conversely, the use of VM on the covariate did not inflate Type I error rate and provided accurate estimates of the effect of the group on the outcome variable.

The progressive shrinking of available conditions when using the VM procedure ensures a perfect balance in the number of participants across conditions while still minimising covariate imbalance. However, some participants will be forcefully assigned to a given condition irrespective of their scores in the covariates. Therefore, in some instances, the researcher will know in advance the condition the participants will be assigned to and not all participants will have the chance to be assigned to each of the available conditions. This restriction might be relevant for clinical trials where one of the conditions is potentially beneficial (i.e., the treatment group). In this case, the researcher can insert a random component into the VM procedure by defining the probability to implement a random assignment. The random component prevents the researcher from being sure about the condition some participants will be assigned to and gives all participants the possibility, in principle, to be assigned to one of the conditions. Using a small amount of randomness (e.g., pRand = 0.1) provides a good balance between matching groups on covariates while avoiding predictable allocation (see section Discontinuous Implementation of the VM Procedure: The Parameter pRand, in the Supplementary Materials).

Despite the benefits of the minimisation procedure, limitations must be carefully considered. First, the application of the VM procedure on small sample sizes does not prevent the treatment effect from being influenced by the unequal distribution of unobserved confounding variables, whose equal distribution is most likely achieved with large sample sizes. This limitation related to small sample sizes affects both the VM procedure and random assignment. Nevertheless, the selection of matching covariates for the minimisation procedure encourages researchers to carefully think in advance about possible confounding variables and match participants on them. Secondly, we showed that the VM is beneficial in simple ANOVA/ANCOVA simulations. In the case of more complex models (e.g., with an interaction), the researcher should carefully consider whether the minimisation procedure constitutes an advantage to the design. We recommend running simulations tailored to specific research designs to ensure that the VM procedure adequately matches participants across conditions.

Third, the minimisation procedure considers all covariates equally important without giving the user the possibility to allow more imbalance in some covariates compared to others (for a minimisation procedure that allows weighting see Saghaei, 2011). It is therefore paramount that the researchers will carefully consider the covariates they wish to match the groups on.

Overall, our minimisation procedure, even after considering the above-mentioned limitations, provides important advantages over the randomisation procedure that is used frequently. Its relative simplicity encourages researchers to use covariate-adaptive matching procedures (Ciolino et al., 2019; Lin et al., 2015). To allow the requested shift from the randomisation procedure, we provide scripts, written using popular software (i.e., R, Python, MATLAB, and Excel), which allow a fast and easy implementation of the VM procedure and integration with other stimulus presentation and analysis scripts. In this light, the treatment can start in the same session in which pre-treatment measures are acquired, thereby reducing the total number of sessions and, consequently, the overall costs. The immediate application of the treatment also excludes the possibility that pre-treatment measures change between the period of the initial recruitment and the actual implementation of the treatment. We strongly recommend using the VM procedure in these studies to yield more effective and valid RCTs.

## Supplementary Information

ESM 1 (DOCX 2855 kb)
