# Correction: When randomisation is not good enough: Matching groups in intervention studies

**DOI:** 10.3758/s13423-026-02910-x

**Published:** 2026-04-02

**Authors:** Francesco Sella, Gal Raz, Roi Cohen Kadosh

**Affiliations:** 1 Centre for Mathematical Cognition, Loughborough University, Loughborough, UK; 2 Department of Experimental Psychology, University of Oxford, Oxford, UK; 3 School of Psychology, University of Surrey, Guildford, United Kingdom

**Correction: Psychonomic Bulletin & Review (2021) 28:2085-2093**

10.3758/s13423-021-01970-5

Correction and Update to Sella et al. (2021)

Juliane Nagel^1^ · Roi Cohen Kadosh^2^ · Francesco Sella^3^

1 Department of Clinical Psychology, Central Institute of Mental Health, Medical Faculty Mannheim, University of Heidelberg, Mannheim, Germany

2 School of Psychology, University of Surrey, UK

3 Centre for Mathematical Cognition, School of Science, Loughborough University, UK

Contribution: JN identified a mismatch between the description of the variance minimisation (VM) procedure in the original paper and its actual implementation in R. JN corrected the code, and together with FS revised the VM function and ran the simulations. FS drafted the manuscript. All authors reviewed and approved the final version of the manuscript.

**Corresponding author**:

Francesco Sella

Centre for Mathematical Cognition, School of Science, Loughborough University, LE11 3TU, Leicestershire, UK

Email: f.sella@lboro.ac.uk

**Correction**

We acknowledge that the description of the variance minimisation (VM) procedure in our published article does not fully match the implementation provided (Sella et al., 2021, p. 2086-2087). Specifically, while we stated that the algorithm minimises the between-group variance by calculating the standard deviation of matching variable means across all groups, the actual implementation calculates this value only across the subset of groups with the lowest number of participants – i.e., the ones currently eligible for assignment. This distinction, though minor, means that the implemented algorithm seeks to minimise variance locally rather than globally. We regret that the implemented algorithm did not align with the original concept and apologise for this oversight.

Despite this difference, the original VM procedure remains robust and continues to outperform random assignment across a range of scenarios, as shown in the original paper (Sella et al., 2021).

**Update**

Here, we present the updated VM procedure that minimises the between-group variance by calculating the standard deviation of matching variable means across all groups, as originally intended.

To assess the difference between the two versions, we ran a simulation based on the first example in the original paper, comparing: the original VM procedure (which considers only eligible groups); an updated version (which calculates variance across all groups); and random assignment. The updated version of the function, along with the simulation code and results, is openly available on the Open Science Framework (OSF) at the following link: https://osf.io/b7wkz.

**Simulation scenario (replicated from original paper)**

In the fictional example, a researcher wants to evaluate whether the combination of cognitive training of executive functions and brain stimulation improves the clinical symptoms of ADHD. The study design comprises three groups: the first group receives brain stimulation and executive functions training; the second group receives sham stimulation and the training; the third group receives neither training nor stimulation. The researcher aims to match the three groups on intelligence, executive function performance, attentional performance, and gender. We simulated 1,000 data sets by randomly drawing scores for IQ, executive functions, and attentional performance from a normal distribution (*M* = 100, *SD* = 15), and gender from a binomial distribution (*p* = .5). Sample sizes were set to *n* = 36, 66, 159, and 969, corresponding to extremely large, large, medium, and small expected effect sizes (*f* = 0.55, 0.40, 0.25, 0.10), with alpha = .05 and power = 80% (Faul et al., 2009). Participants were assigned using random assignment, the original VM procedure, and the updated VM procedure. For each data set, we ran ANOVAs on IQ, executive functions, and attentional performance, and χ2 tests on gender.

**Results**

Figure 1 shows the distributions of F, p, and η2 values (top panel) and χ2, p, and Cramer’s V (bottom panel). Both VM versions outperformed random assignment. Although the updated version is more strongly optimised to minimise variance across groups, it shows no clear performance advantage over the original. Going beyond the simulations reported in the original paper, we computed the population standard deviation across groups after each assignment of an individual participant. After every assignment step, we compared whether the original or the updated procedure currently leads to the lowest variance between groups (omitting the first three participants which were assigned to one of the empty groups). The original procedure performed worse than the updated procedure in 45.22% of assignment steps. The updated procedure performed worse than the original procedure in 45.01% of assignment steps. The two procedures produced identical between-group variances in 9.77% of assignment steps. Thus, the updated procedure was overall better at optimising the between-group variance but did not always produce better assignments than the original procedure, which we discuss below.

**Fig. 1:**
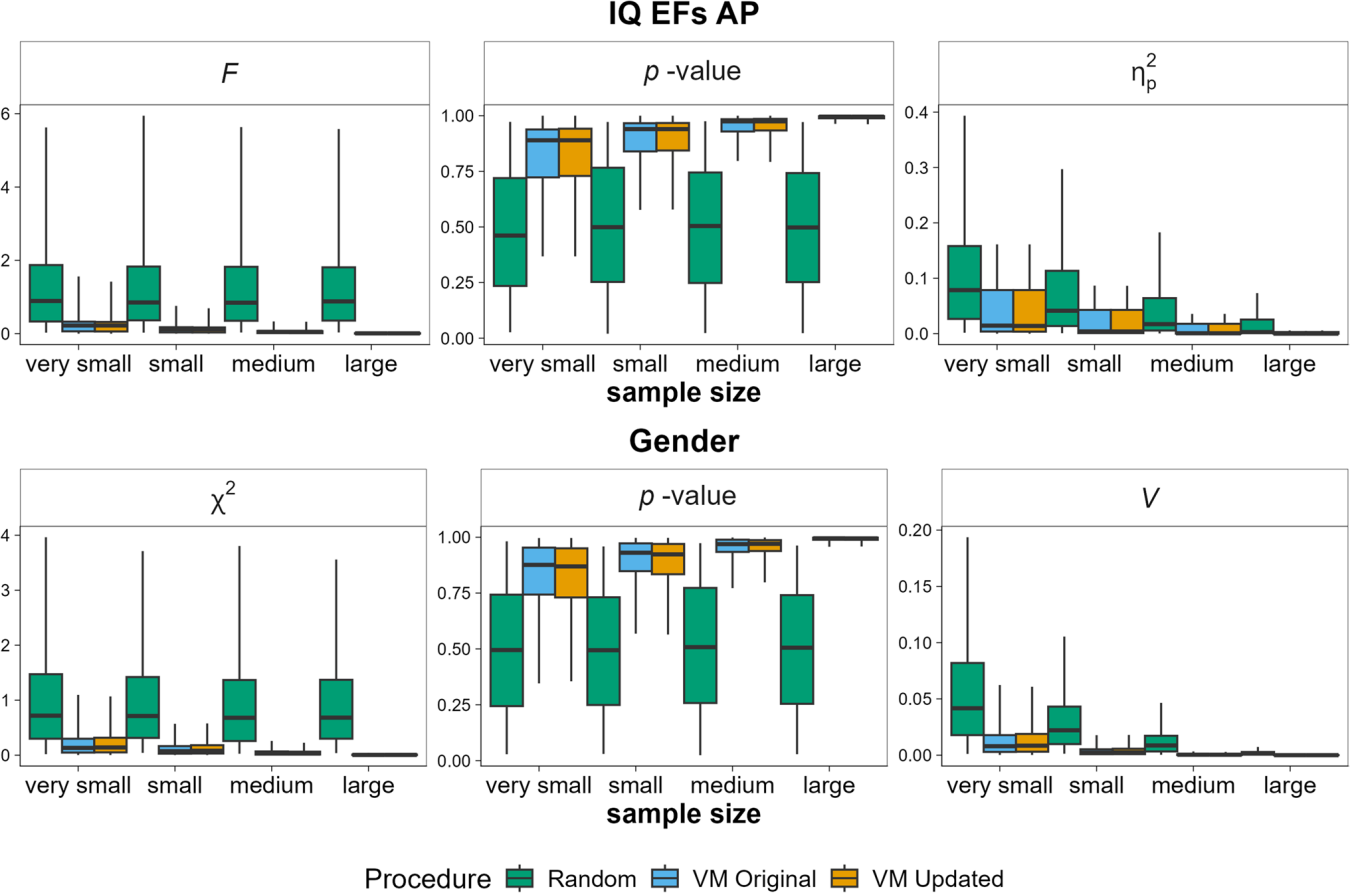
A comparison of the updated VM procedure (orange boxplot), the original VM procedure (blue boxplot) and random assignment (green boxplot) based on simulated data. Top panel: Distributions of F-values, p-values, and η2 values from ANOVAs comparing groups on intelligence (IQ), executive functions (EFs), and attentional performance (AP). Bottom panel: Distributions of χ2, p-values, and Cramer’s V values comparing groups on gender. The boxplots represent the quartiles whereas the whiskers represent the 95% limits of the distribution

**Comparison under equal conditions**

Given that we fixed an error in the implementation, one might expect the updated version to clearly outperform the original version of the VM procedure. The reason why this was not the case is that in any stepwise VM procedure, future assignments are dependent on earlier assignments: A previous optimal assignment may prevent a future optimal assignment. This limitation masks the improvements of the updated VM procedure. To demonstrate that the updated VM procedure indeed outperforms the original version, we compared the two functions under identical conditions, i.e., when previous assignments were identical. To this end, we utilised the data generated in the previous simulation. We used the group assignments created by the random, updated VM and original VM assignment, and re-assigned the second-to-last participant (ignoring the data of the last participant). We then compared the between-group variance after re-assigning the second-to-last participant using both the original and updated VM procedure. We only compared assignments of the second-to-last participant, because in the simulated data sets, the sample size is divisible by the group size. For instance, with 36 participants, every group assignment (group 1–3) occurs equally often. When re-assigning the last participant, only one of the groups has the minimum number of participants, resulting in a deterministic assignment. Likewise, when the 34th participant is assigned, all three groups will be considered, in which scenario the outcome for the original and the updated VM procedure will be identical. However, when re-assigning the 35th participant, the original VM procedure will wrongly only minimise the variance across the two groups considered for an assignment, while the updated VM procedure will take into account the variance across all three groups. Thus, we compared the two procedures in a scenario where differences are in principle possible. Under these conditions, the original and the updated VM procedure produced identical between-group variances (due to identical assignments) in 92.58% of cases. In 7.42%, the updated procedure outperformed the original procedure. The original procedure never outperformed the updated procedure. This demonstrates that the updated procedure is better at optimising the target metric (reduced between-group variance). However, it also becomes apparent that the original and updated procedure produce identical results in the majority of cases. In our simulations, the percentage of identical assignments is even over-estimated, because we initially discard two-thirds of cases where the two procedures would definitely produce identical results.

**Conclusion**

In summary, although the original VM procedure minimises variance locally – by considering only the groups eligible for assignment – it still performs well and as expected. The R, Excel, MATLAB and Python implementations provided with the original paper remain valid and can continue to be used considering both the original findings and the present simulation. However, the updated version of the function, which calculates variance across all groups as originally described, is now available in R (https://osf.io/b7wkz). Researchers may consider using this revised version, as it appears to offer a slight improvement in minimising between-group differences.

