# Supplementary material for: When randomisation is not good enough: Matching groups in intervention studies
Source: Psychon Bull Rev. 2021 Jul 9;28(6):2085–93. doi: 10.3758/s13423-021-01970-5 (PMC8642369; doi:10.3758/s13423-021-01970-5)
Supplement: Supplementary file 1 — (DOCX 2855 kb) [file 13423_2021_1970_MOESM1_ESM.docx]

**Supplementary materials**

**1. Details of the minimisation procedure**

The problem is to assign each observation to the group that will minimise group differences while keeping the number of participants in each group balanced. The procedure works as follows:

1. The first participants joining the study are sequentially assigned one to each group. That is, if there are *K* groups, then the first *K* participants will be directly allocated to the groups 1, 2,…,*K,* respectively.
2. Let *n* be the number of already assigned participants, i.e. after step 1), *n* equals *K*. The (*n+*1)^th^ participant is temporarily assigned to one group at a time, and for each assignment, the algorithm converts raw scores of each variable *p* into standardised scores *x_p_*, by subtracting the mean and dividing by the standard deviation across groups.
3. Next, we calculate the mean of each variable in each group for each possible group assignment of our current participant. Let $m_{p}^{k}(j)$ denote the mean of all observations of the *p*^th^ variable in the *k*^th^ group, given that the (n+1)^th^ participant, is temporarily assigned to the *j*^th^ group, such that:

$$m_{p}^{k}\left( j \right)=\{\frac{1}{\left| G_{k} \right|} \sum_{i\in G_{k}} x_{p}^{i} if k\neq j \frac{1}{\left| G_{k} \right|+1}\sum_{i\in G_{k}\cup\{n+1\}} x_{p}^{i} if k=j,$$

where *G_k_* denotes the set of indices of participants in the *k*^th^ group, and |*G_k_*| refers to the number of participants allocated to that group.

1. From this, we can compute the mean of means between groups for the variable *p,* given that the (*n*+1)^th^ observation is added to group *j:*

$$\mu_{p}\left( j \right)= \frac{1}{K}\sum_{k} m_{p}^{k}(j)$$

2. Then, we compute the sum of squared deviations (SS) of the group means of the overall$\mu_{p}$of each variable *p*, given the current assignment of the (*n*+1)^th^ participant to group *j*:

$$SS(j)=\sum_{p} \sum_{k} \left( m_{p}^{k}\left( j \right)-\mu_{p}\left( j \right) \right)^{2}$$

3. Given that we now have a statistic capturing how much variance there is in the group means for each possible label for the (*n*+1)^th^ participant, we assign this observation to the group label *j* that minimises this statistic:

$$label\left( n+1 \right)=SS\left( j \right) ,$$

where *S* is the set of groups to which the (*n+1*)^th^ participant could have been allocated.

The next participant undergoes the same procedure from step 2 onwards, but the algorithm will assign the current participant to one of the groups that previous participant/s were not assigned to. That is, the set *S* shrinks by the group label *j* that satisfied the variance minimisation procedure in step 6). The same procedure goes on until *S* contains only one remaining group label, to which the current participant is then automatically assigned. Then, the entire procedure starts again from the beginning with the possibility for the next participant to be assigned to all groups, i.e. *S =* {1, 2,…,*K*}.

**2. Assessing ANOVA assumptions**

We assessed the assumption of normality of residuals and homogeneity of variance across sample sizes for the first ANOVA reported in the main text (i.e., matching on IQ, EFs, AP and gender). We ran Shapiro tests on the residuals of the models and Levene tests on the dependent variable with group as a factor. In Figure S1, we reported the percentage of significant (*p*<.05) Shapiro and Levene tests across sample sizes. The VM procedure and the random assignment violated the normality of residuals at a similar percentage. The VM procedure violated normality of the residuals slightly more often in the case of large sample sizes whereas it appears to produce fewer violations of homogeneity of variance in the case of very small sample sizes compared to the random assignment.


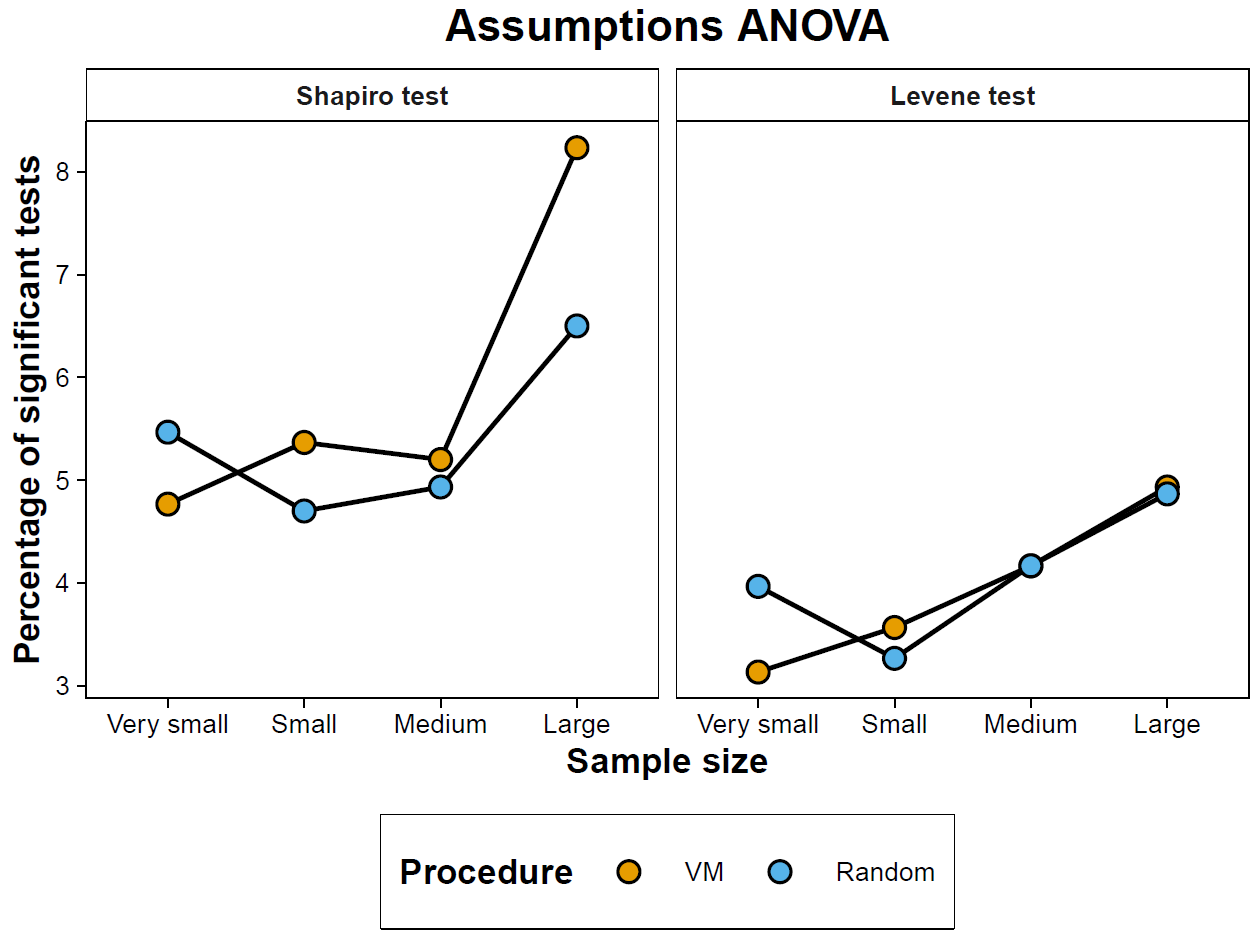


Figure S1. Percentage of significant (*p*<.05) Shapiro and Levene tests in the ANOVA simulations separately for VM procedure (orange dots) and random assignment (blue dots).

**3. Additional simulations**

We added two simulations to demonstrate two additional features of the VM procedure. In the first simulation, we show how the user can set a parameter, “pRand”, to decide the probability of the VM procedure to be replaced with random assignment. This random component ensures that the assignment of participants is not completely determined by the VM procedure. In the second simulation, we show how the VM procedure can efficiently match participants on non-dichotomous nominal variables when these are expressed as dummy variables.

**3.1 Discontinuous implementation of the VM procedure: the parameter pRand**

The VM procedure allocates the present participant to the condition that minimises the imbalance in the covariates while keeping track of previous allocations. For instance, if there are three conditions (i.e., A, B, and C) and the first participant was assigned to group B, and the second to group A, the third participant, independently of the scores on covariates, will be assigned to group C. In this way, the number of participants in each condition is kept balanced while minimisation is implemented. One might have a concern that the forced assignment enables the researcher to reliably predict the condition the next participant will be assigned to. Another concern is that only a few participants have the opportunity to be assigned to all the available conditions, some of which could be potentially beneficial (i.e., new treatment vs placebo).

Our approach overcomes these issues by giving the user the possibility to specify the probability to implement random assignment over the VM procedure. The user can set this probability by varying the parameter “pRand” between 0 and 1, where 0 means that the VM procedure is always implemented (as done in the previous simulation) and 1 means that participants are randomly assigned to conditions. This random component prevents the assignment of participants to be totally determined based on participant’s scores and on previous allocations. Therefore, a participant, irrespective of their score on the matching covariates, always has the chance to be assigned to all conditions.

We matched participants on a hypothetical randomly distributed IQ score (*M*=100, *SD*=15) by using the VM procedure with “pRand'' set to 0 (VM procedure is always implemented), 0.1 (VM procedure is implemented approximately 90% of the times), 0.5 (50% of the times), and 1 (never; i.e., random assignment) in 1000 simulations. As in our previous simulation, we varied the sample size to be very small (n=36), small (n=66), medium (n=159), and large (n=969) reflecting the researcher’s intention to evaluate the possible presence of an extremely large (f=0.55), large (f=0.40), medium (f=0.25), and small (f=0.10) effect size respectively, while keeping the alpha at .05 and power at 80% (Faul et al., 2009). In Figure 3, we reported the distributions of *F*, *p*, and η^2^ values from ANOVAs on IQ. As expected, we noted a progressive increase in *F* and η^2^ values and a tendency of the distribution of *p*-values to become less skewed toward 1 when we passed from always implementing the VM procedure to implementing it approximately 90% of times (i.e., pRand=0.1), 50% of times (i.e., pRand=0.5), or never (i.e., pRand=1; random assignment).


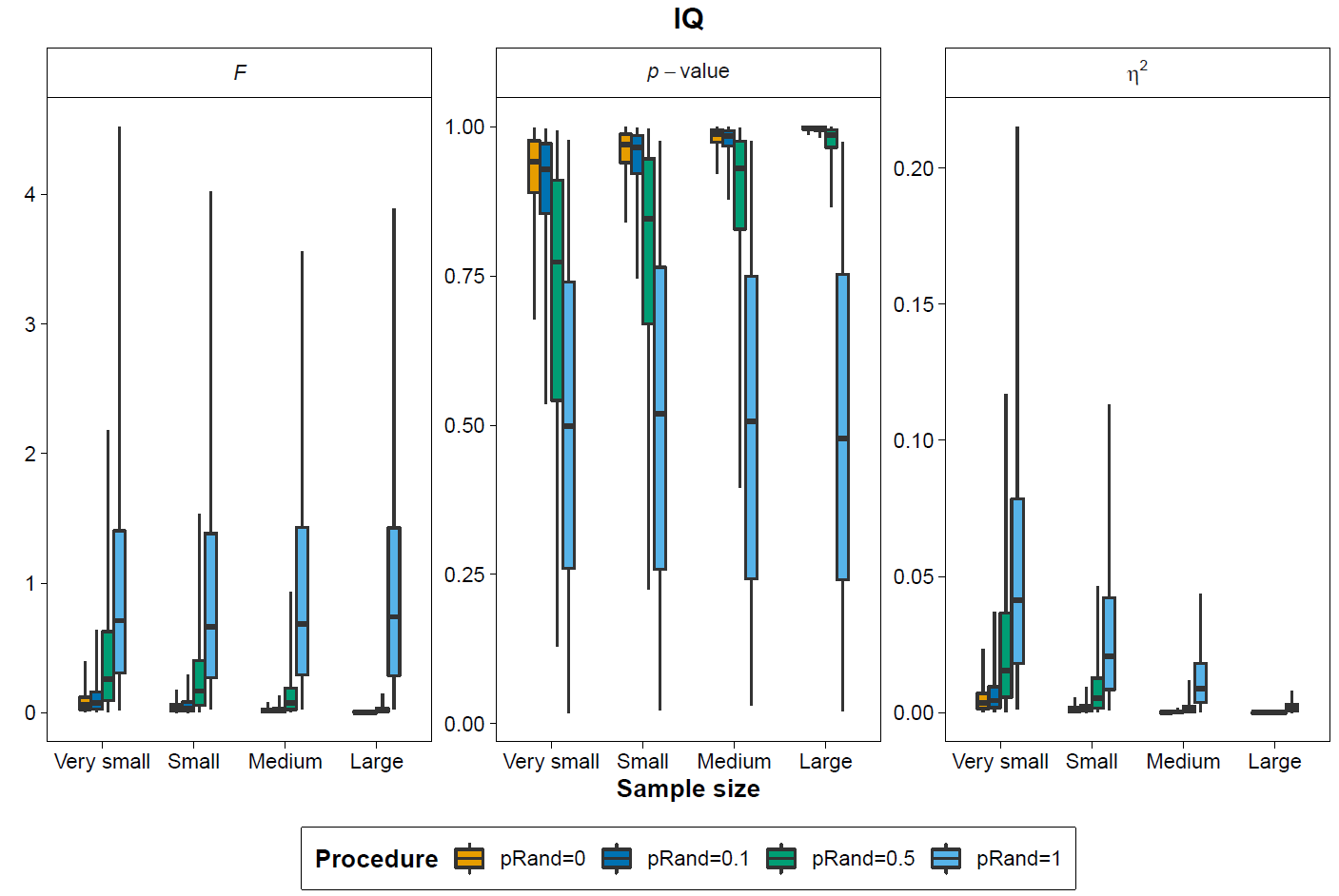


Figure S2. Distributions of *F*-values, *p*-values, and η^2^ values from ANOVAs comparing groups on intelligence (IQ) separately for different values of the pRand parameter in the VM procedure. The boxplots represent the quartiles whereas the whiskers represent the 95% limits of the distribution.

The discontinuous implementation of the VM procedure (e.g., setting pRand>0) may cause some imbalance in the number of participants that are assigned to each condition. Note that always applying the VM (i.e., pRand=0) or randomly assigning participants in advance would lead to a perfect balance of the number of participants in each condition (assuming that the total number of participants is a multiple of the number of conditions). We estimated the mean percentage imbalance in the number of participants in each condition when the pRand was set to 0.1, 0.5, and 1. In our simulation, having three conditions, we would expect to have 33% of participants in each condition. Then, we calculated the absolute difference between the observed percentage and the expected one. For instance, if a condition had 32% of participants, the imbalance would be 1%. Finally, we calculated the mean percentage of absolute imbalance for the different values of pRand separately for each sample size (i.e., very small=36; small=66; medium=159; large=969). The mean percentage imbalance was negligible when pRand was 0.1 and 0.5 and more pronounced when pRand was 1. However, the imbalance became smaller with increasing sample sizes (pRand=0.1: very small=0.64%; small=0.33%; medium=0.14%; large=0.02%; pRand=0.5: very small=4.27%; small=2.15%; medium=0.91%; large=0.14%; pRand=1: very small=19.1%; small=13.5%; medium=9.09%; large=3.59%).

In order to prevent forced assignment, the user can manipulate the probability of implementing the VM procedure, rather than using random assignment, by setting the parameter pRand. The less frequent use of the VM procedure (i.e. high values of pRand) reduces its effectiveness in matching participants on covariates and minimally compromises the accuracy in keeping the number of participants in each condition balanced.

**3.2 Using VM procedure on non-dichotomous nominal variables**

The VM procedure successfully matches conditions on binary categorical variables (e.g., male/female as reported in the first simulation) as it considers those variables to be numerical (i.e, 0 and 1). However, the VM procedure cannot directly implement a matching based on categorical/nominal covariates with more than two levels. Nevertheless, the user could overcome this restriction by transforming the categorical variable(s) in dummy variables (i.e., 0 or 1). For example, a researcher assigns participants who have received one of three diagnoses (i.e., the categorical variable with three levels: D1, D2, and D3) to two treatment conditions. The categorical variable diagnosis is transformed in three dummy variables (i.e., D1, D2, and D3) whereby participants with a diagnosis of D1 would get a 1 in the D1 variable and a 0 in the D2 and D3 variables. Similarly, participants with a diagnosis of D2 would get 1 in the D2 variable and a 0 in the D1 and D3 variables. Participants with a diagnosis of D3 would get 1 in the D3 variable and a 0 in the D1 and D2 variables. We compared the distribution of the three diagnoses (i.e., D1, D2, and D3) in the two treatment conditions when using the VM procedure or random assignment in 1000 simulations. We varied the sample size to be very small (n=20), small (n=40), medium (n=108), and large (n=964) reflecting the researcher’s intention to evaluate the possible presence of an extremely large (f=0.70), large (f=0.50), medium (f=0.30), and small (f=0.10) effect size respectively, while keeping the alpha at .05 and power at 80% (Faul et al., 2009). We randomly sampled each of the three diagnoses with the same probability until the selected sample size was reached. In Figure 4, we show the distributions of χ^2^, *p*, and Cramer’s *V* values on the contingency table entailing diagnosis and condition. The χ^2^ and Cramer’s *V* values tended to be small whereas the distribution of *p*-values was skewed toward 1, rather than uniform, when we implemented the VM procedure compared to random assignment. The VM procedure demonstrated an efficient matching on categorical variables between conditions starting from a very small sample size. However, the user must be aware that nominal variables with several levels may become predominant in matching procedure. Accordingly, the VM procedure will match the groups on several dummy variables, that are expressions of a single nominal variable, at the expense of other matching variables.


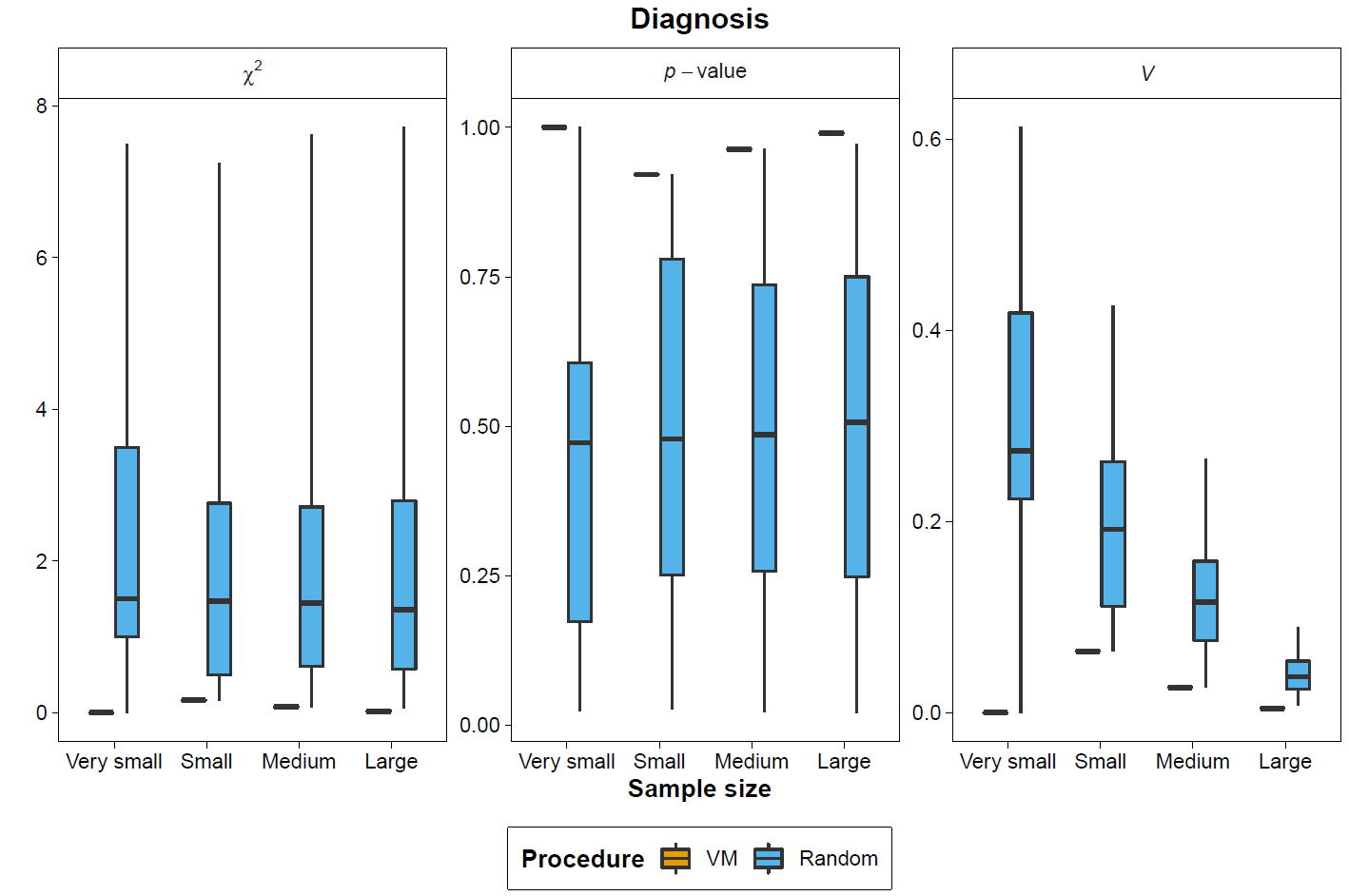


Figure S3. Distributions of χ^2^, *p*-values, and Cramer’s *V* values comparing groups on diagnosis separately for the VM procedure (red boxplots) and the random assignment (lilac boxplots). The boxplots represent the quartiles whereas the whiskers represent the 95% limits of the distribution.

**4. Patterns of correlations of the covariate B in the ANCOVA simulation**

| *Correlation pattern CovB* | *Procedure* | *Correlation* | *median* | *ll95%* | *ul95%* |
| --- | --- | --- | --- | --- | --- |
| Cor pattern CovB 1 | Random | Y - CovB | 0.29 | -0.16 | 0.62 |
|  |  | CovA - CovB | 0.50 | 0.23 | 0.73 |
|  | VM on CovA | Y - CovB | 0.29 | -0.17 | 0.62 |
|  |  | CovA - CovB | 0.50 | 0.24 | 0.73 |
| Cor pattern CovB 2 | Random | Y - CovB | 0.50 | 0.22 | 0.72 |
|  |  | CovA - CovB | 0.28 | -0.16 | 0.61 |
|  | VM on CovA | Y - CovB | 0.50 | 0.22 | 0.72 |
|  |  | CovA - CovB | 0.29 | -0.16 | 0.61 |
| Cor pattern CovB 3 | Random | Y - CovB | 0.00 | -0.30 | 0.30 |
|  |  | CovA - CovB | 0.00 | -0.30 | 0.30 |
|  | VM on CovA | Y - CovB | -0.00 | -0.30 | 0.30 |
|  |  | CovA - CovB | 0.00 | -0.30 | 0.30 |

Table S1. Median and 95% lower and upper limits of the distribution of correlation between the covariate A (CovA), the covariate B (CovB) and the dependent variable Y in three correlation patterns of the covariate B (e.g., 1, 2, 3) separately for the random assignment and the minimisation procedure.

**5 Assessing ANCOVA assumptions**

We assessed the assumption of normality of residuals and homogeneity of variance in the ANCOVA scenario reported in the main text.We did not control for homogeneity of slopes as the correlation between covariates and the dependent variable was set to be similar across groups. We ran shapiro tests on the residuals of the models and levene tests on the dependent variable with group as factor. In Figure S2, we reported the percentage of significant (*p*<.05) Shapiro and Levene tests. Note that in the case of ANCOVA we collapsed the results coming from the three patterns of correlations of the covariate B and the presence or absence of the treatment effect. The VM procedure and the random assignment violated the normality of residuals at a similar percentage. The VM procedure and the control for covariate approach violated normality of the residuals and homogeneity of variance with a similar rate.


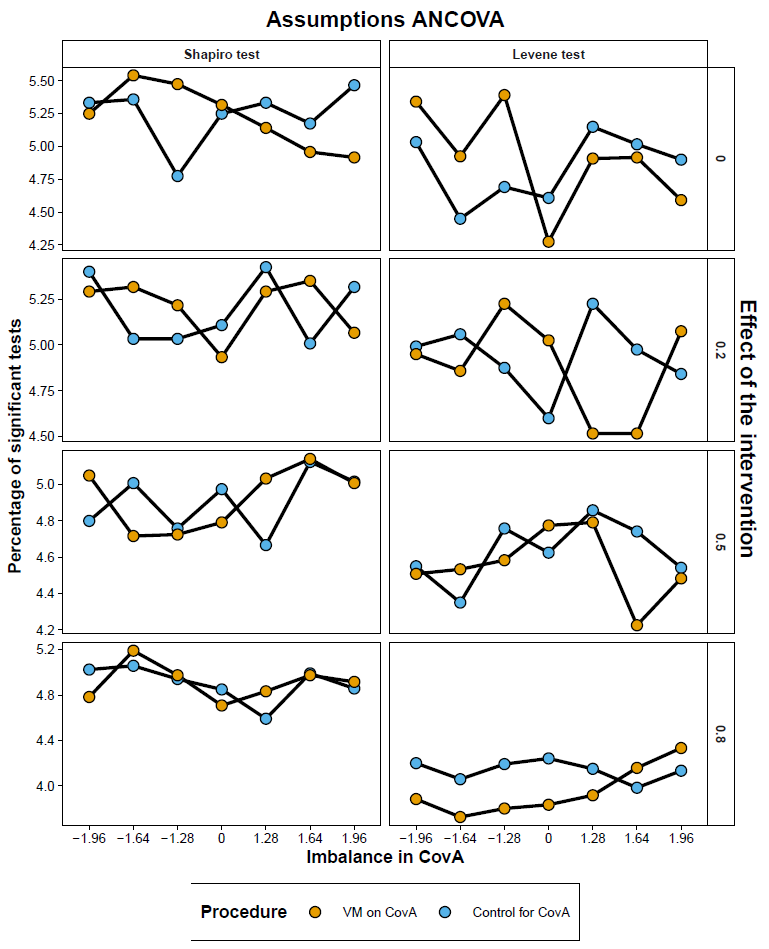


Figure S4. Percentage of significant (*p*<.05) Shapiro (left-panel) and Levene tests (right-panel) in the ANCOVA simulation as a function of imbalance in the covariate A (x-axis) across the effect size to be detected (rows; absent=0, n=788; small=0.2, n=788; medium=0.5, n=128; large=0.8, n=52) separately for VM procedure (orange dots) and random assignment (blue dots).

**6. Proportion of significant results and estimated effect for the covariate A, B, and group in the ANCOVA for the different patterns of correlations of CovB.**


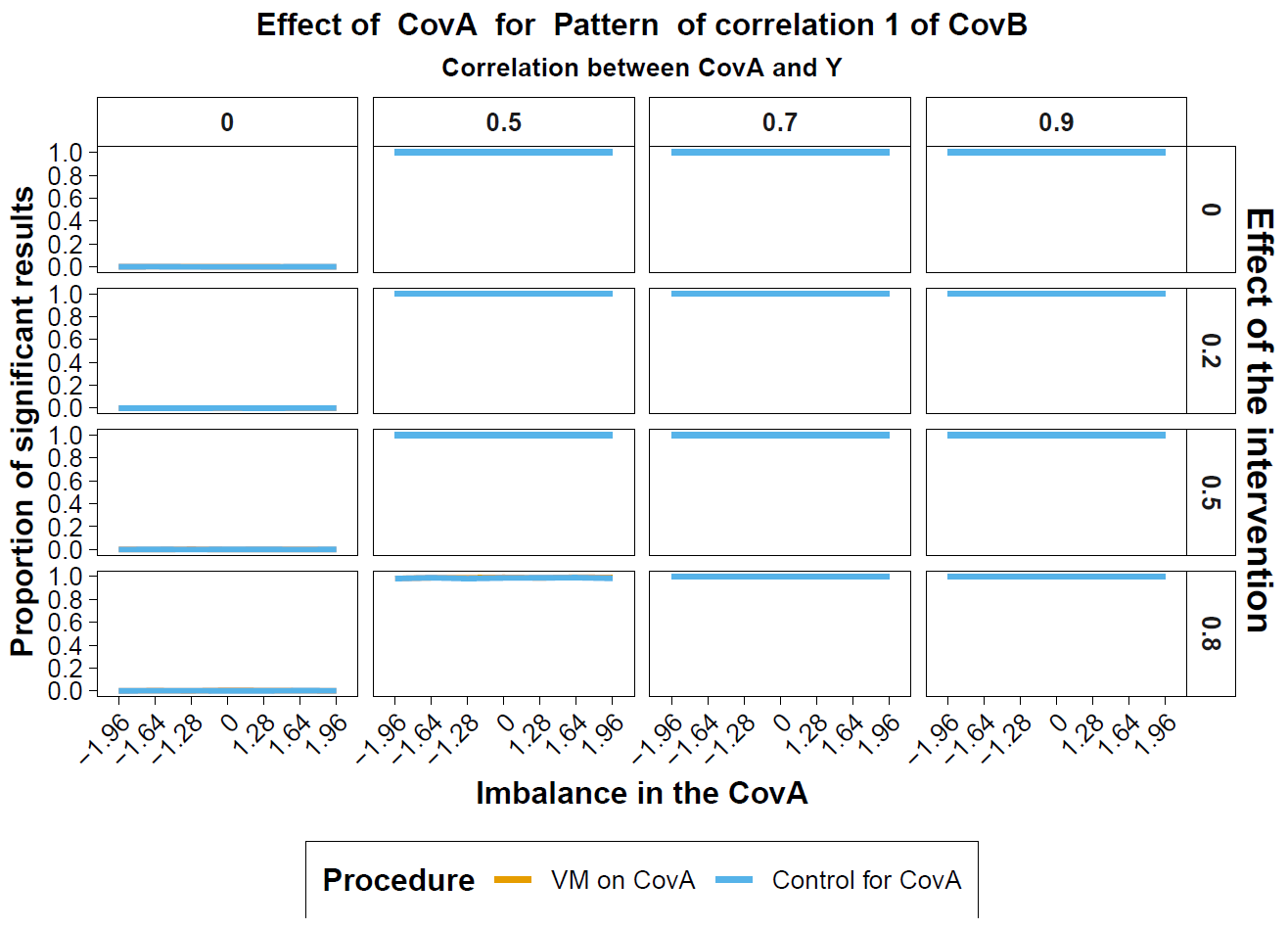


Figure S5. Proportion of significant results (y-axis) for the effect of CovA for pattern of correlation 1 of CovB in the ANCOVA (Y ~ CovA + CovB + Group) separately for the VM procedure (orange lines) and control for CovA approach (blue lines) across imbalances of the covariate A (x-axis) when the sample size varied according to the effect size to be detected (rows; absent=0, n=788; small=0.2, n=788; medium=0.5, n=128; large=0.8, n=52) and the correlation between the covariate A and the dependent variable Y ranged between 0 and 0.9 (columns).


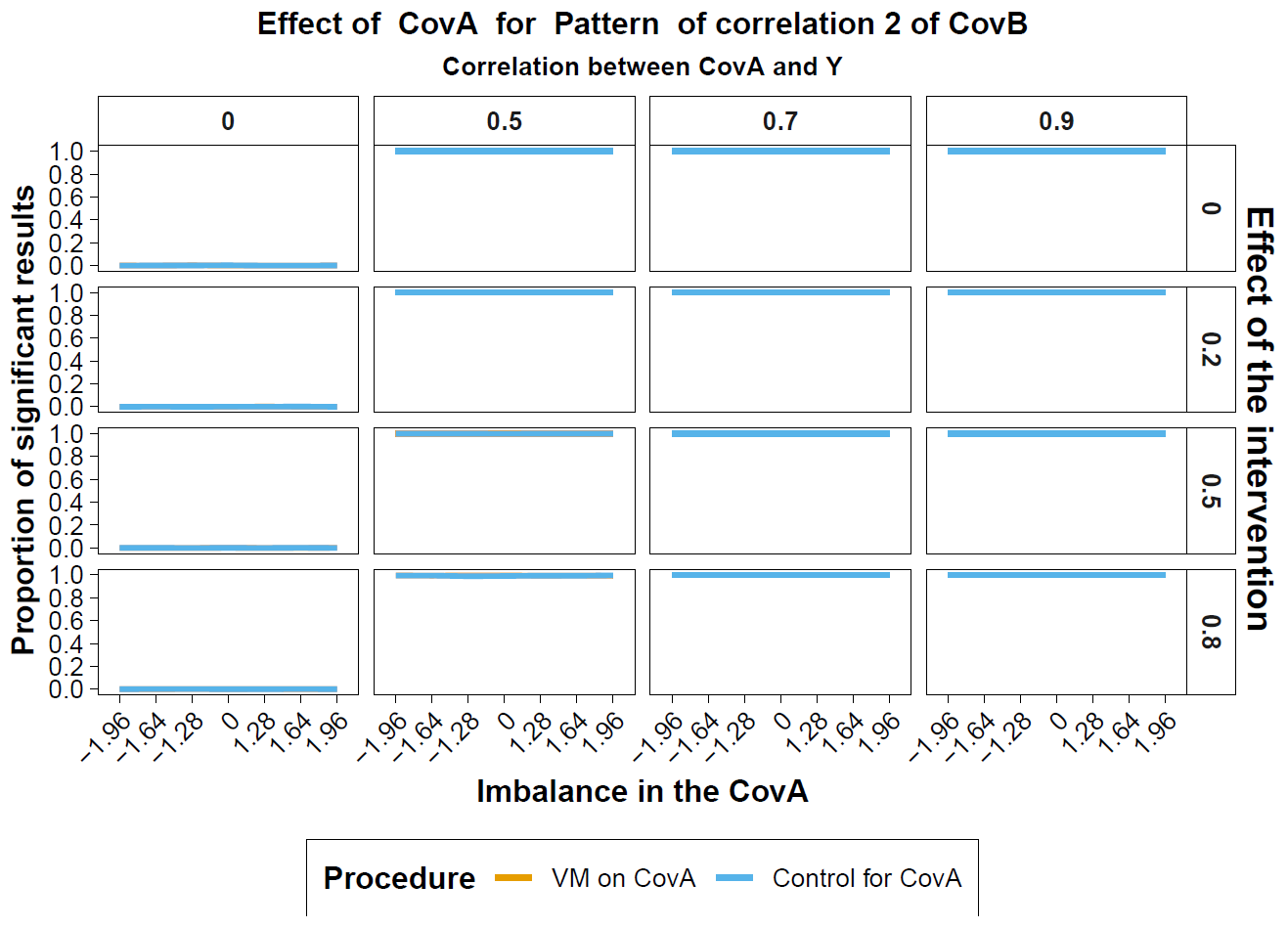


Figure S6. Proportion of significant results (y-axis) for the effect of CovA for pattern of correlation 2 of CovB in the ANCOVA (Y ~ CovA + CovB + Group) separately for the VM procedure (orange lines) and control for CovA approach (blue lines) across imbalances of the covariate A (x-axis) when the sample size varied according to the effect size to be detected (rows; absent=0, n=788; small=0.2, n=788; medium=0.5, n=128; large=0.8, n=52) and the correlation between the covariate A and the dependent variable Y ranged between 0 and 0.9 (columns).


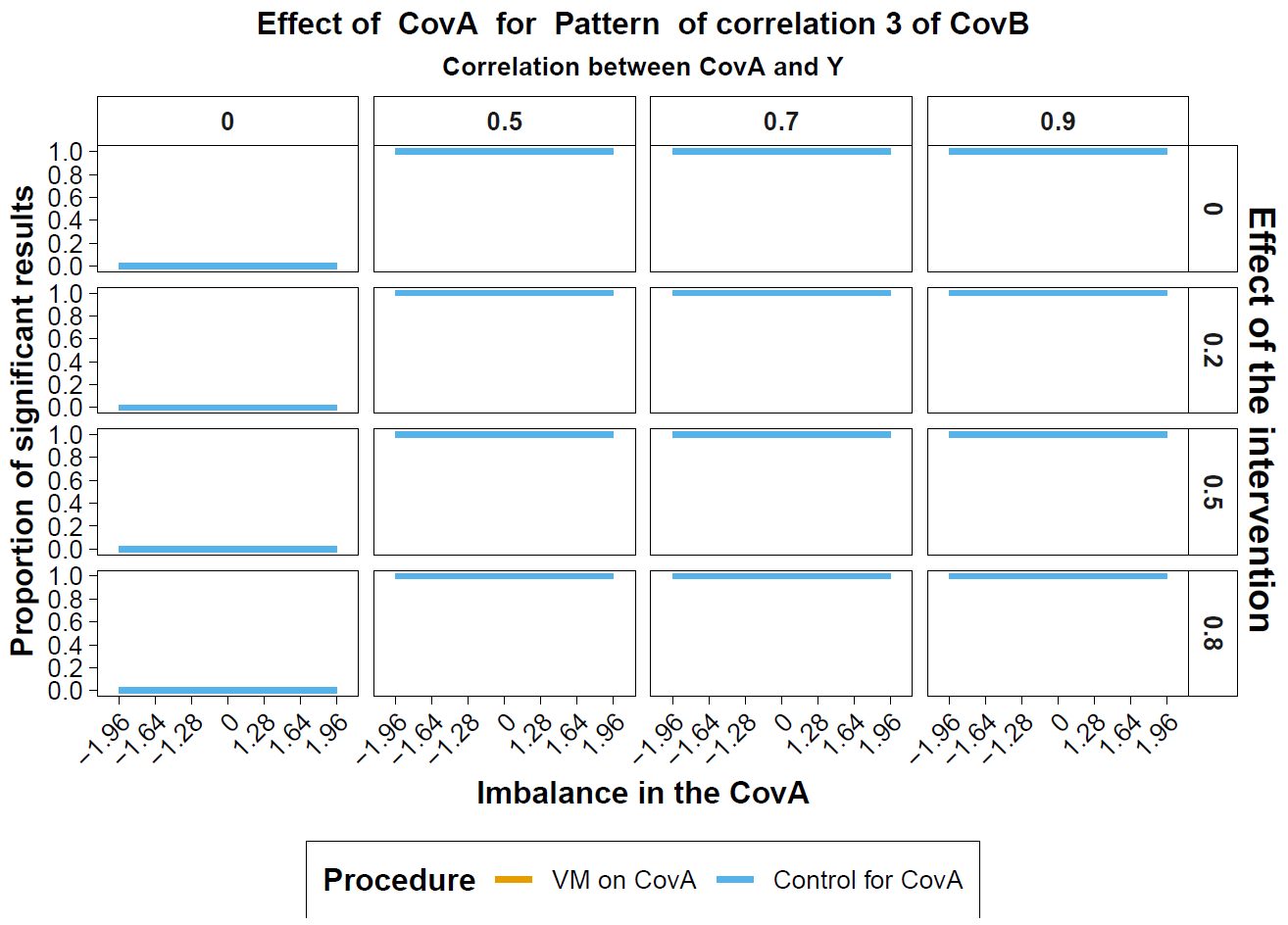


Figure S7. Proportion of significant results (y-axis) for the effect of CovA for pattern of correlation 3 of CovB in the ANCOVA (Y ~ CovA + CovB + Group) separately for the VM procedure (orange lines) and control for CovA approach (blue lines) across imbalances of the covariate A (x-axis) when the sample size varied according to the effect size to be detected (rows; absent=0, n=788; small=0.2, n=788; medium=0.5, n=128; large=0.8, n=52) and the correlation between the covariate A and the dependent variable Y ranged between 0 and 0.9 (columns).


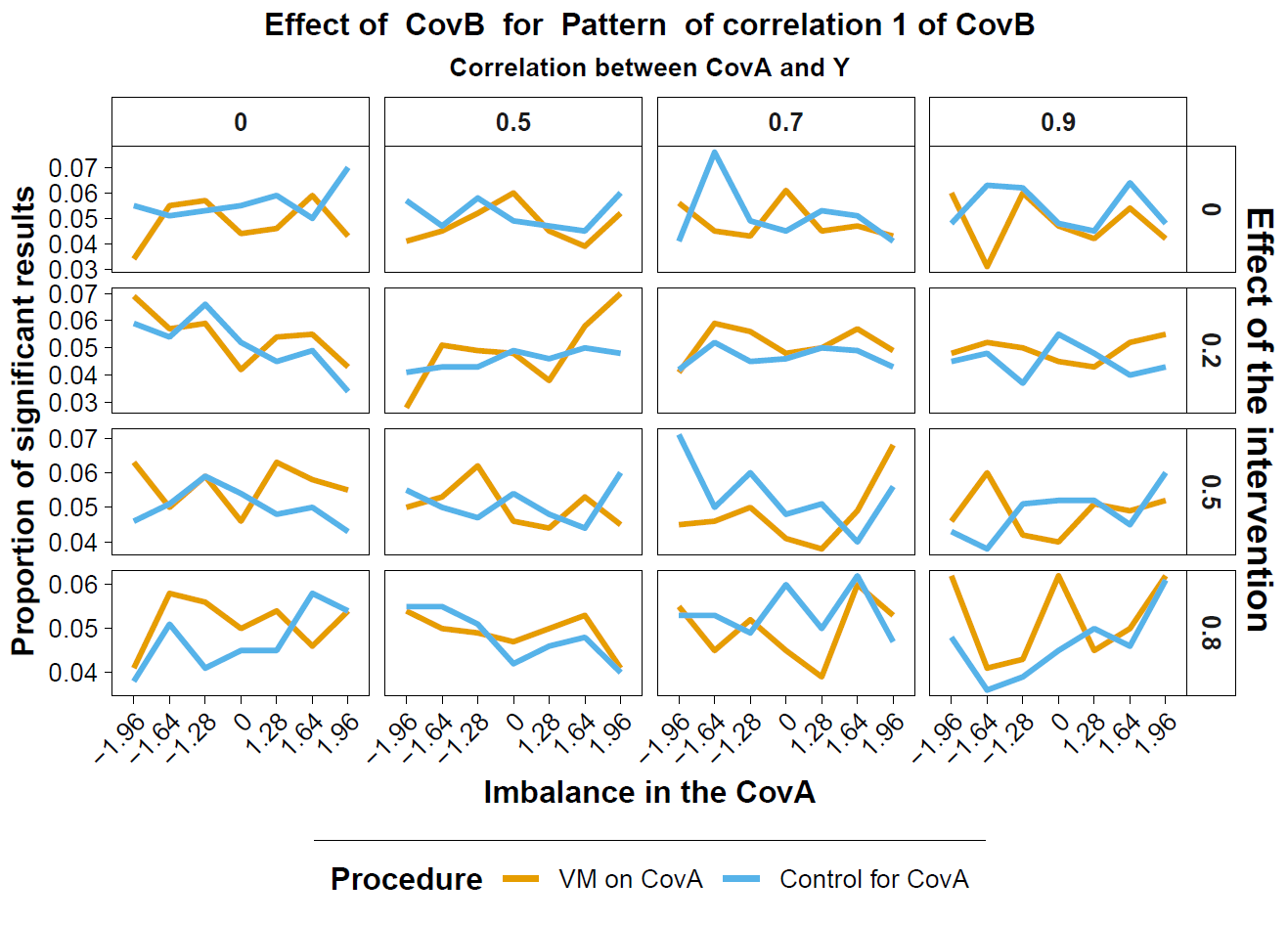


Figure S8. Proportion of significant results (y-axis) for the effect of CovB for pattern of correlation 1 of CovB in the ANCOVA (Y ~ CovA + CovB + Group) separately for the VM procedure (orange lines) and control for CovA approach (blue lines) across imbalances of the covariate A (x-axis) when the sample size varied according to the effect size to be detected (rows; absent=0, n=788; small=0.2, n=788; medium=0.5, n=128; large=0.8, n=52) and the correlation between the covariate A and the dependent variable Y ranged between 0 and 0.9 (columns).


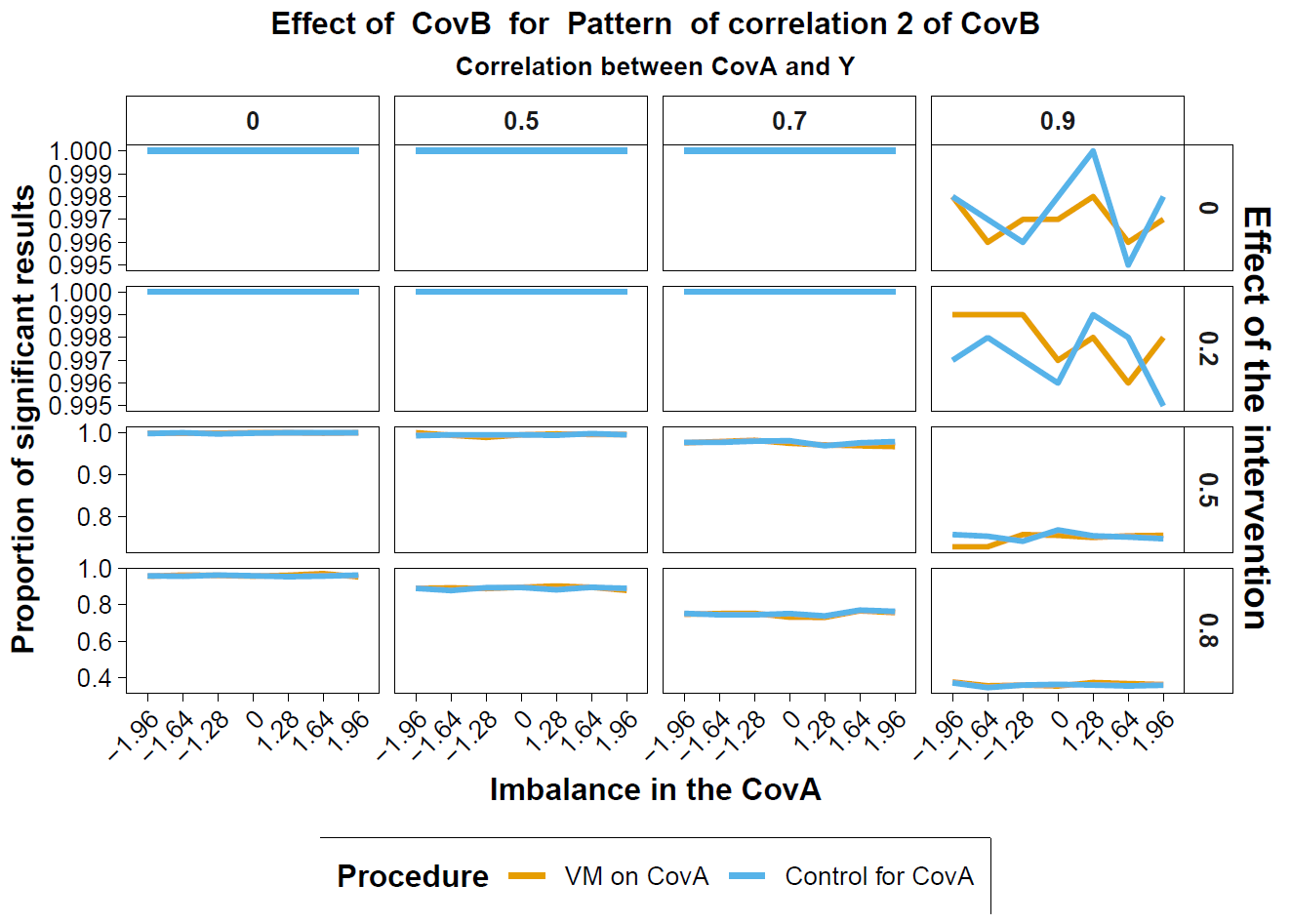


Figure S9. Proportion of significant results (y-axis) for the effect of CovB for pattern of correlation 2 of CovB in the ANCOVA (Y ~ CovA + CovB + Group) separately for the VM procedure (orange lines) and control for CovA approach (blue lines) across imbalances of the covariate A (x-axis) when the sample size varied according to the effect size to be detected (rows; absent=0, n=788; small=0.2, n=788; medium=0.5, n=128; large=0.8, n=52) and the correlation between the covariate A and the dependent variable Y ranged between 0 and 0.9 (columns).


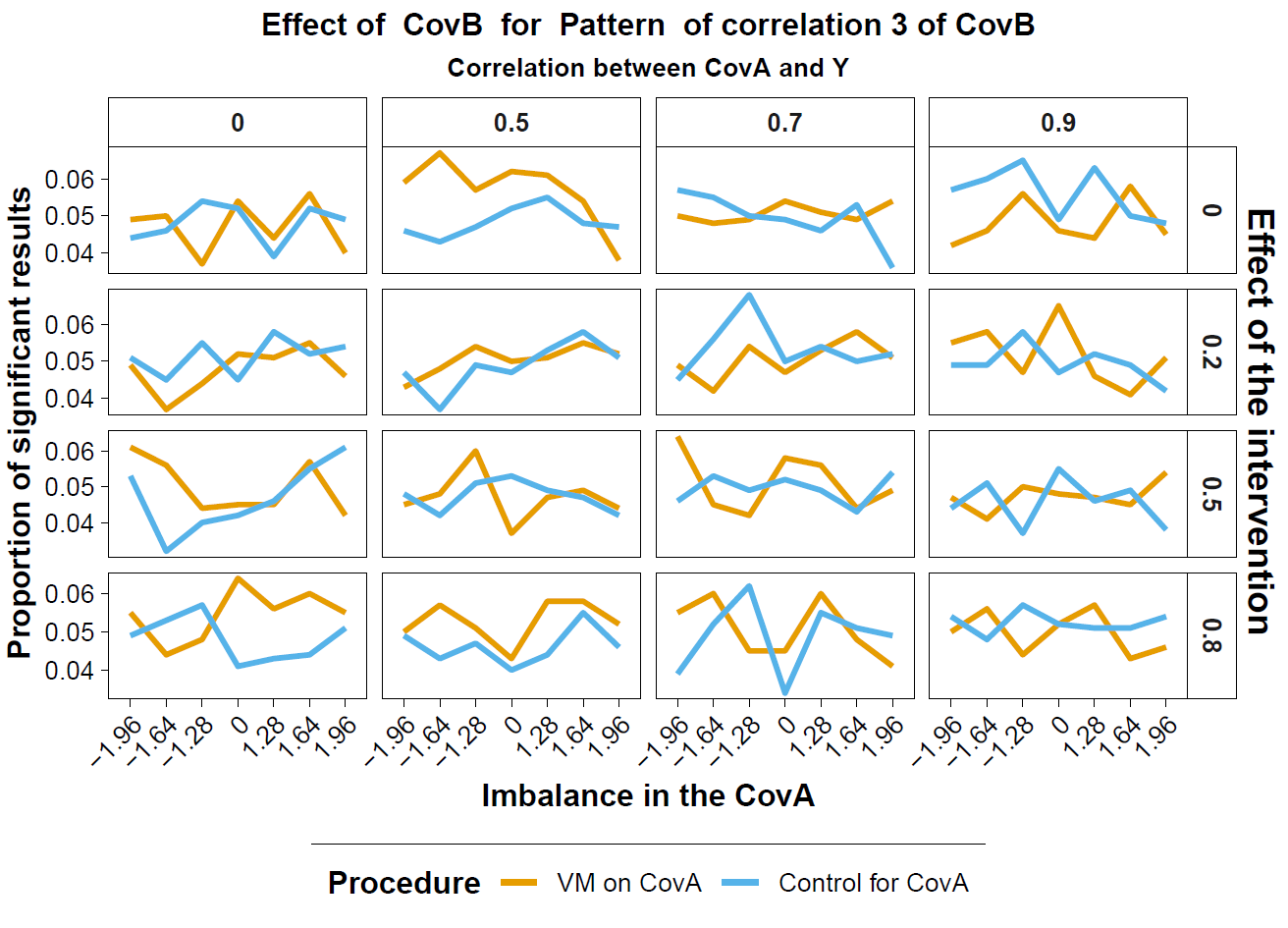


Figure S10. Proportion of significant results (y-axis) for the effect of CovB for pattern of correlation 3 of CovB in the ANCOVA (Y ~ CovA + CovB + Group) separately for the VM procedure (orange lines) and control for CovA approach (blue lines) across imbalances of the covariate A (x-axis) when the sample size varied according to the effect size to be detected (rows; absent=0, n=788; small=0.2, n=788; medium=0.5, n=128; large=0.8, n=52) and the correlation between the covariate A and the dependent variable Y ranged between 0 and 0.9 (columns).


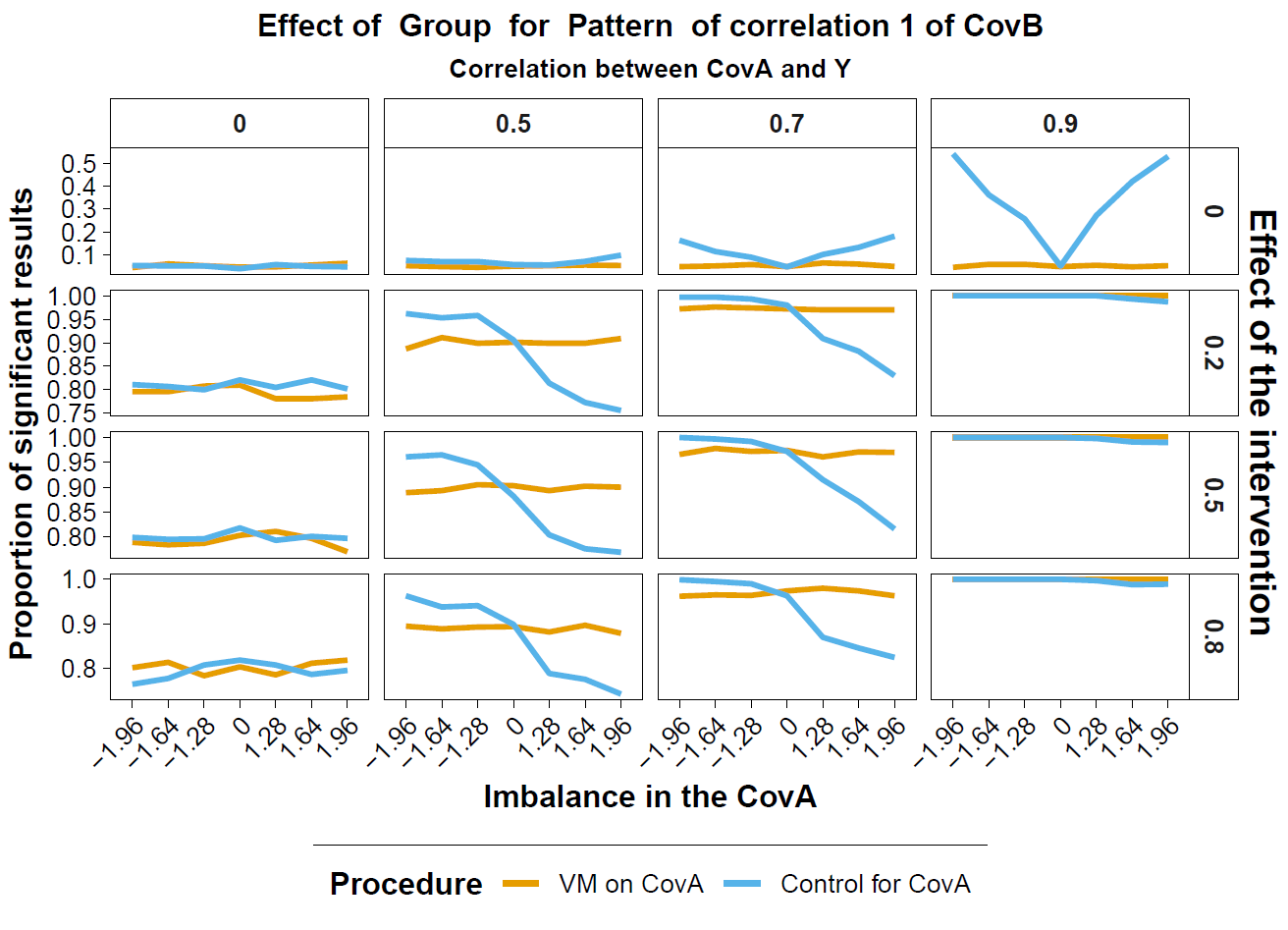


Figure S11. Proportion of significant results (y-axis) for the effect of Group for pattern of correlation 1 of CovB in the ANCOVA (Y ~ CovA + CovB + Group) separately for the VM procedure (orange lines) and control for CovA approach (blue lines) across imbalances of the covariate A (x-axis) when the sample size varied according to the effect size to be detected (rows; absent=0, n=788; small=0.2, n=788; medium=0.5, n=128; large=0.8, n=52) and the correlation between the covariate A and the dependent variable Y ranged between 0 and 0.9 (columns).


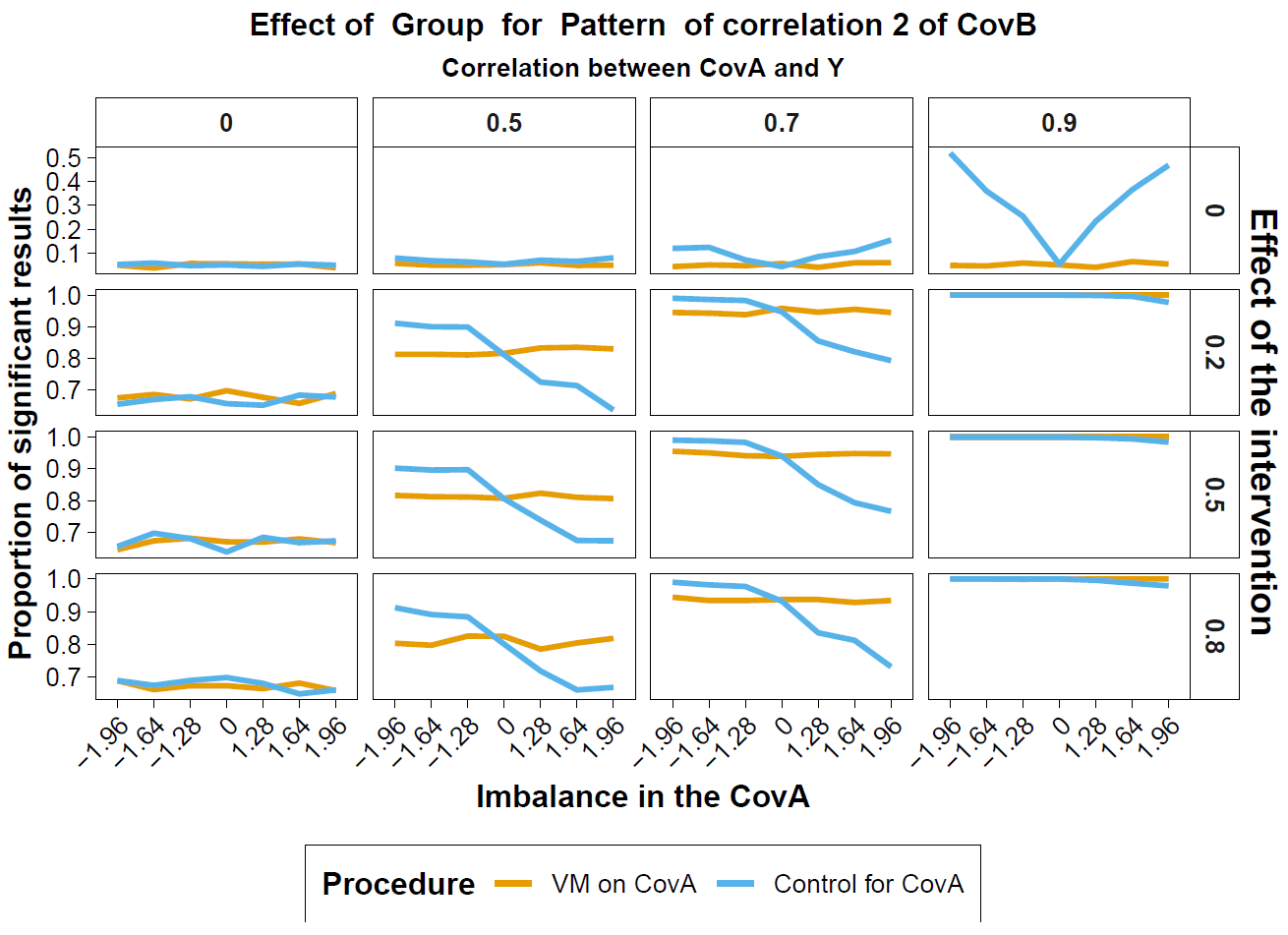


Figure S12. Proportion of significant results (y-axis) for the effect of Group for pattern of correlation 2 of CovB in the ANCOVA (Y ~ CovA + CovB + Group) separately for the VM procedure (orange lines) and control for CovA approach (blue lines) across imbalances of the covariate A (x-axis) when the sample size varied according to the effect size to be detected (rows; absent=0, n=788; small=0.2, n=788; medium=0.5, n=128; large=0.8, n=52) and the correlation between the covariate A and the dependent variable Y ranged between 0 and 0.9 (columns).


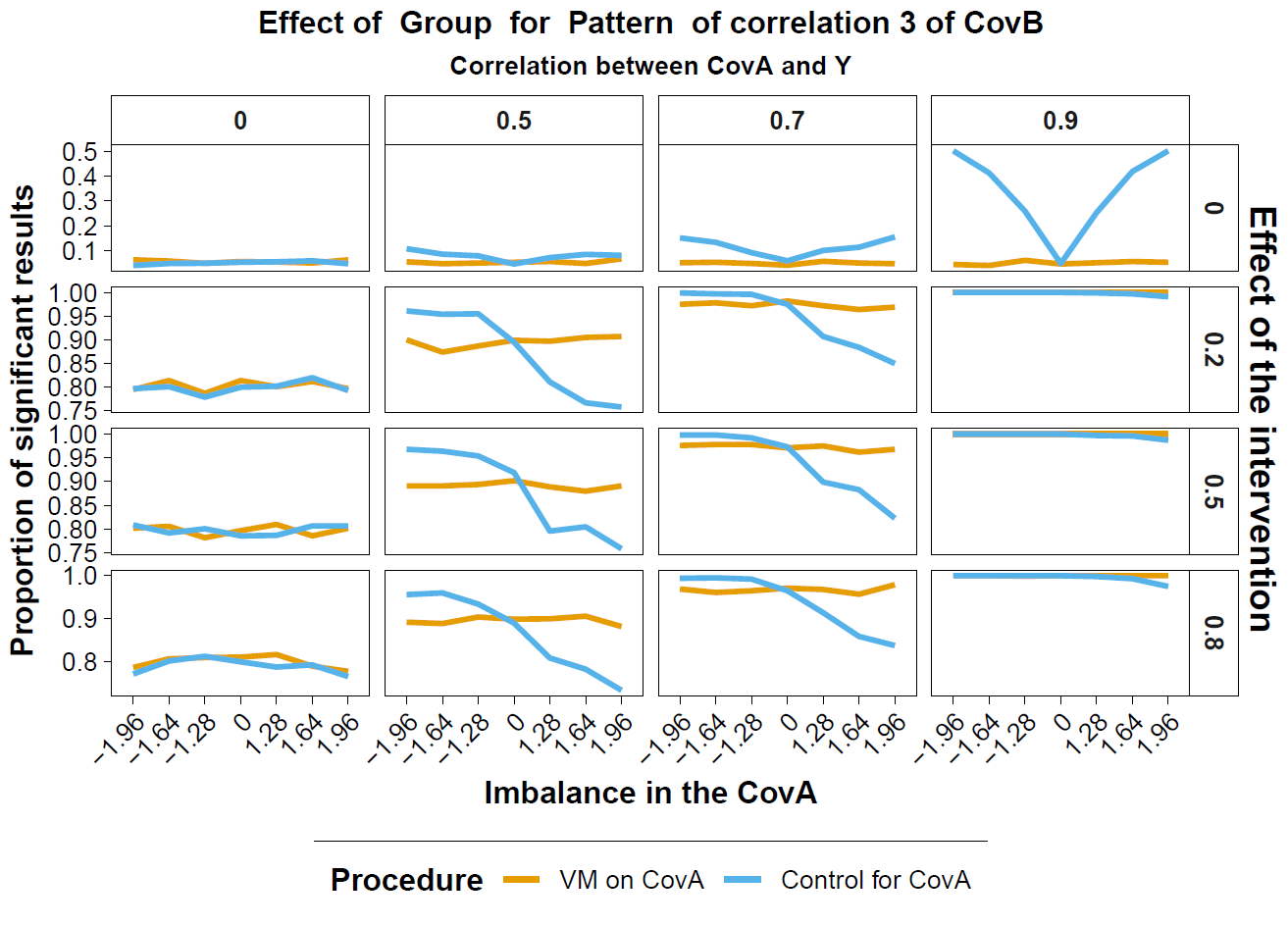


Figure S13. Proportion of significant results (y-axis) for the effect of Group for pattern of correlation 3 of CovB in the ANCOVA (Y ~ CovA + CovB + Group) separately for the VM procedure (orange lines) and control for CovA approach (blue lines) across imbalances of the covariate A (x-axis) when the sample size varied according to the effect size to be detected (rows; absent=0, n=788; small=0.2, n=788; medium=0.5, n=128; large=0.8, n=52) and the correlation between the covariate A and the dependent variable Y ranged between 0 and 0.9 (columns).


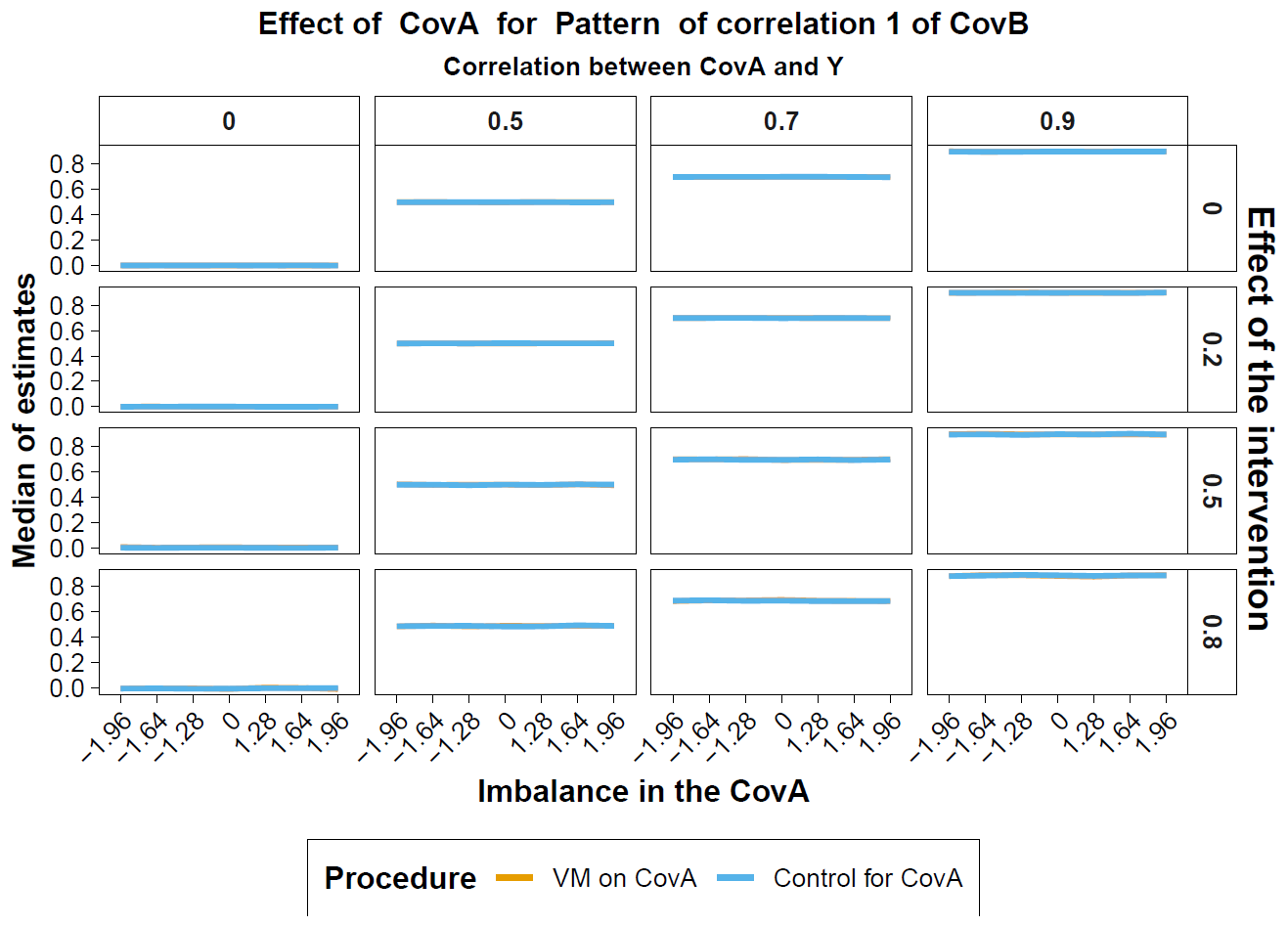


Figure S14. Median of estimates (y-axis; regression coefficients) for the effect of CovA for pattern of correlation 1 of CovB in the ANCOVA (Y ~ CovA + CovB + Group) separately for the VM procedure (orange lines) and control for CovA approach (blue lines) across imbalances of the covariate A (x-axis) when the sample size varied according to the effect size to be detected (rows; absent=0, n=788; small=0.2, n=788; medium=0.5, n=128; large=0.8, n=52) and the correlation between the covariate A and the dependent variable Y ranged between 0 and 0.9 (columns).


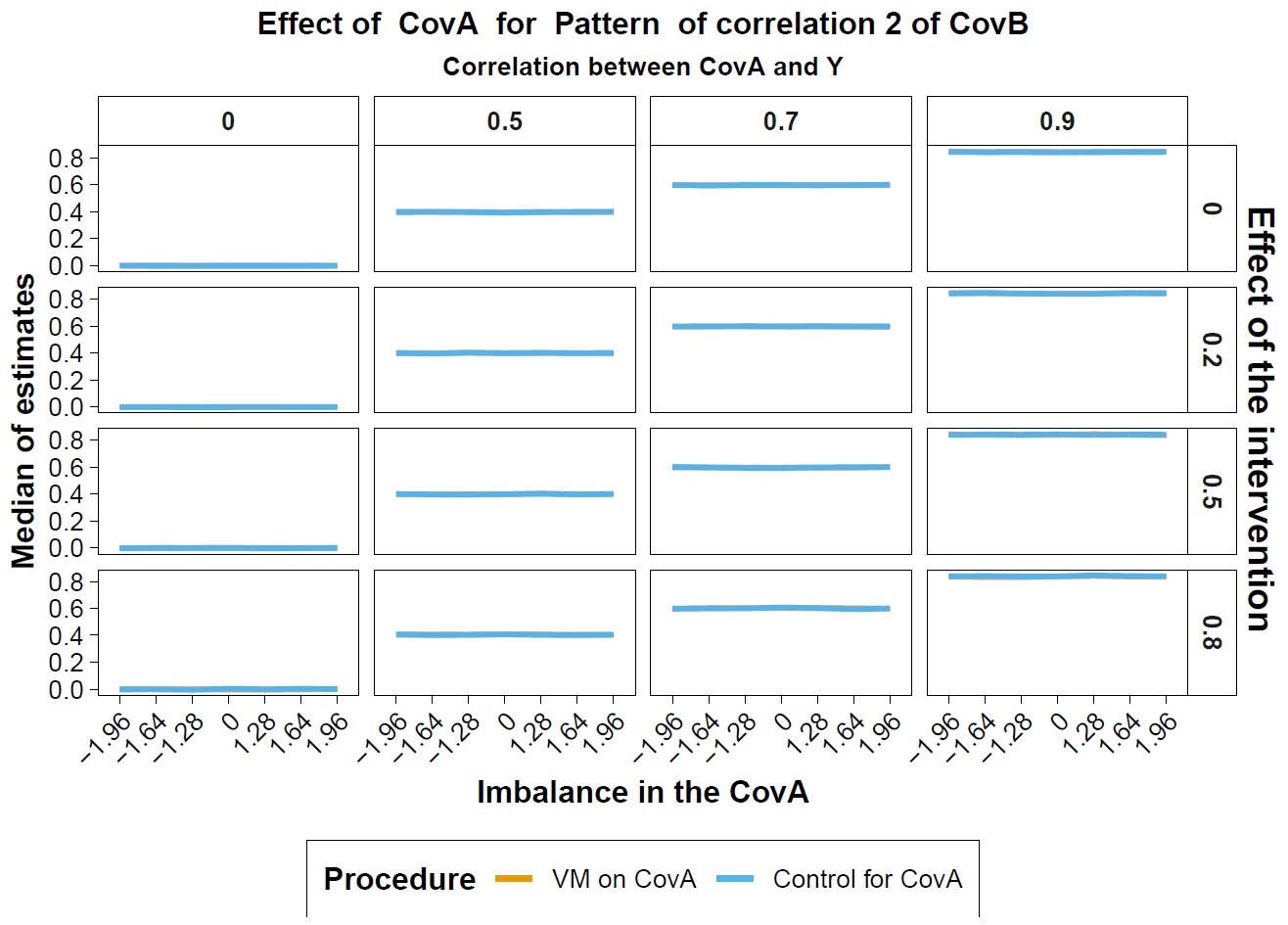


Figure S15. Median of estimates (y-axis; regression coefficients) for the effect of CovA for pattern of correlation 2 of CovB in the ANCOVA (Y ~ CovA + CovB + Group) separately for the VM procedure (orange lines) and control for CovA approach (blue lines) across imbalances of the covariate A (x-axis) when the sample size varied according to the effect size to be detected (rows; absent=0, n=788; small=0.2, n=788; medium=0.5, n=128; large=0.8, n=52) and the correlation between the covariate A and the dependent variable Y ranged between 0 and 0.9 (columns).


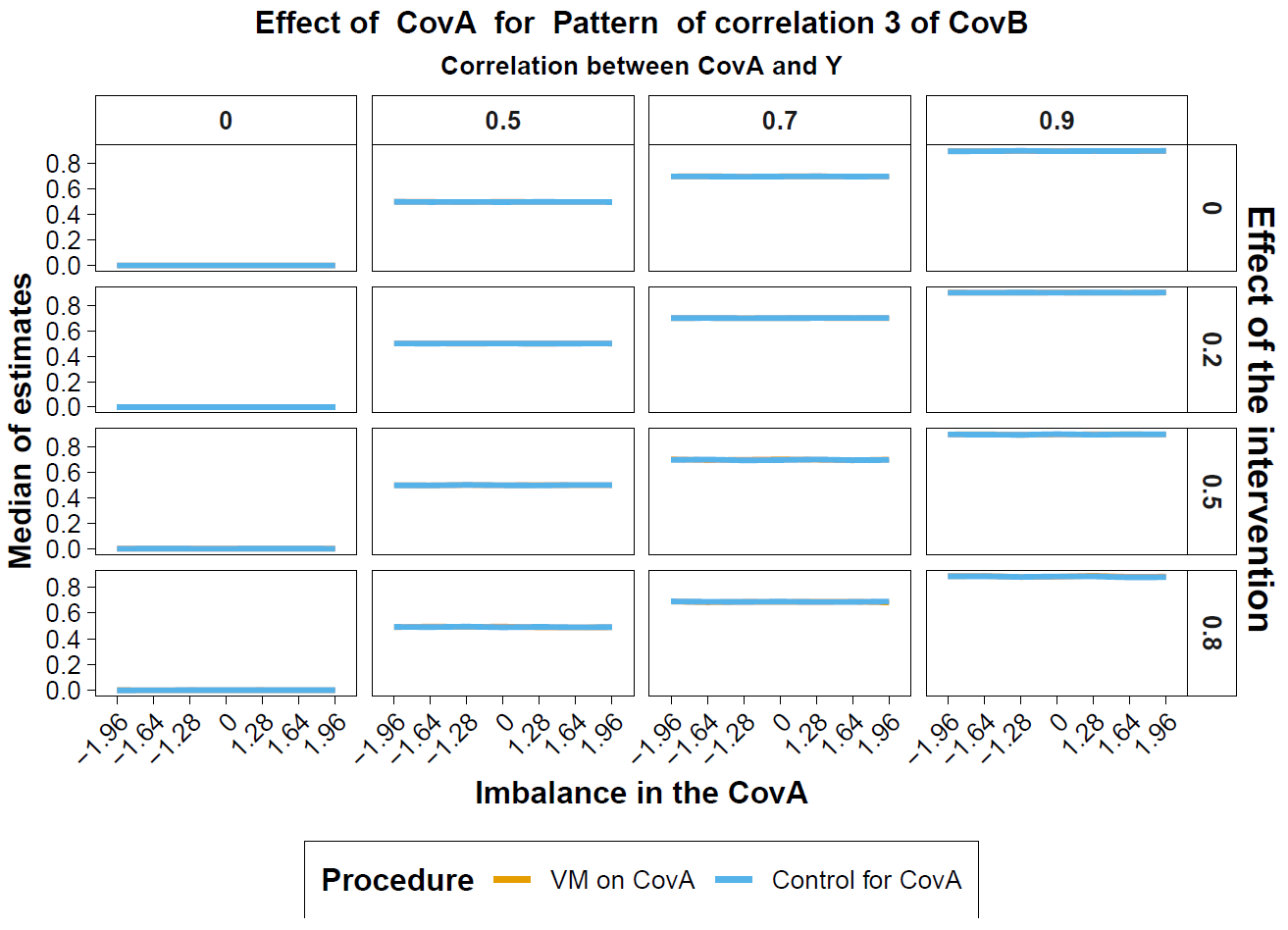


Figure S16. Median of estimates (y-axis; regression coefficients) for the effect of CovA for pattern of correlation 3 of CovB in the ANCOVA (Y ~ CovA + CovB + Group) separately for the VM procedure (orange lines) and control for CovA approach (blue lines) across imbalances of the covariate A (x-axis) when the sample size varied according to the effect size to be detected (rows; absent=0, n=788; small=0.2, n=788; medium=0.5, n=128; large=0.8, n=52) and the correlation between the covariate A and the dependent variable Y ranged between 0 and 0.9 (columns).


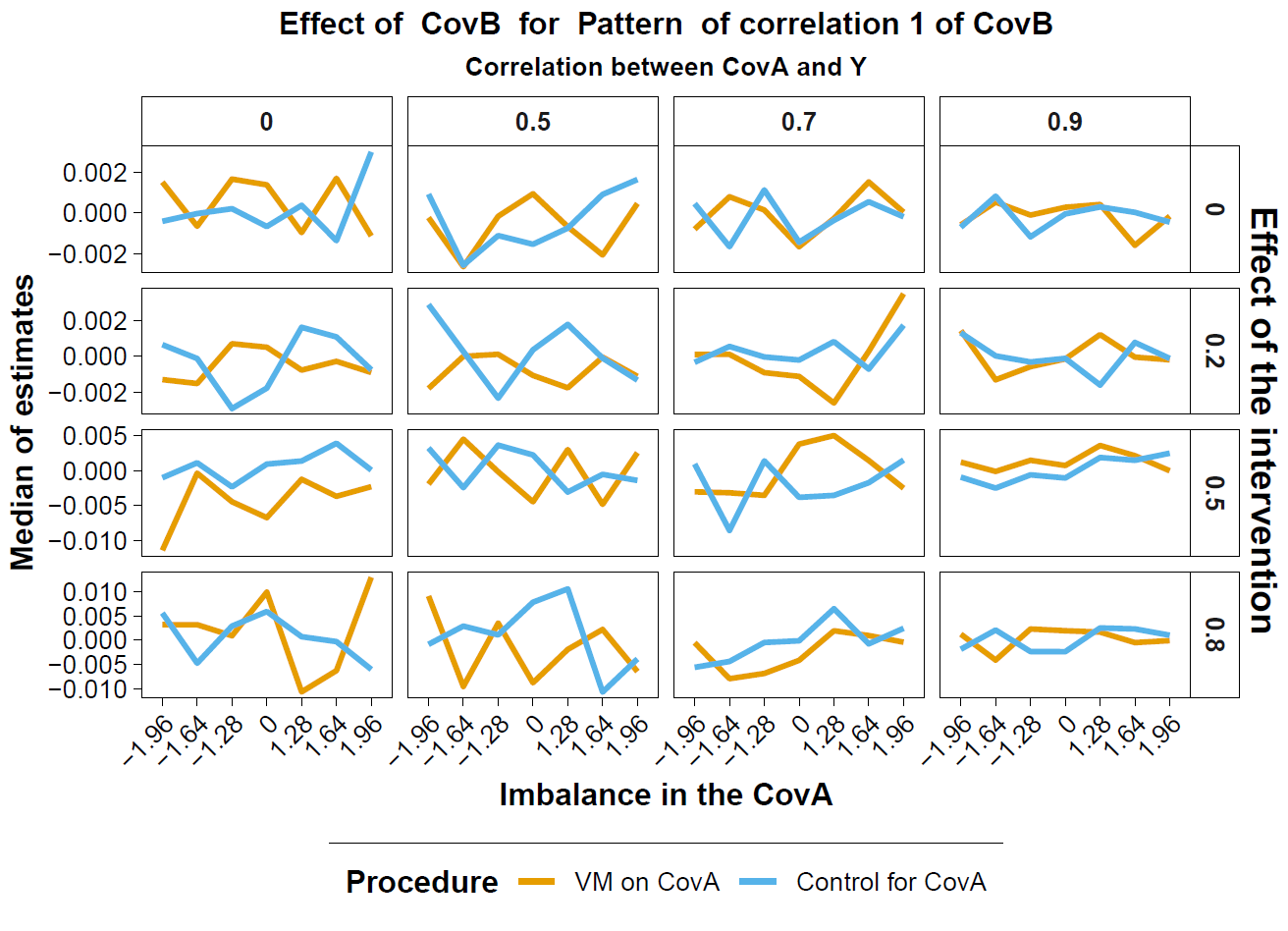


Figure S17. Median of estimates (y-axis; regression coefficients) for the effect of CovB for pattern of correlation 1 of CovB in the ANCOVA (Y ~ CovA + CovB + Group) separately for the VM procedure (orange lines) and control for CovA approach (blue lines) across imbalances of the covariate A (x-axis) when the sample size varied according to the effect size to be detected (rows; absent=0, n=788; small=0.2, n=788; medium=0.5, n=128; large=0.8, n=52) and the correlation between the covariate A and the dependent variable Y ranged between 0 and 0.9 (columns).


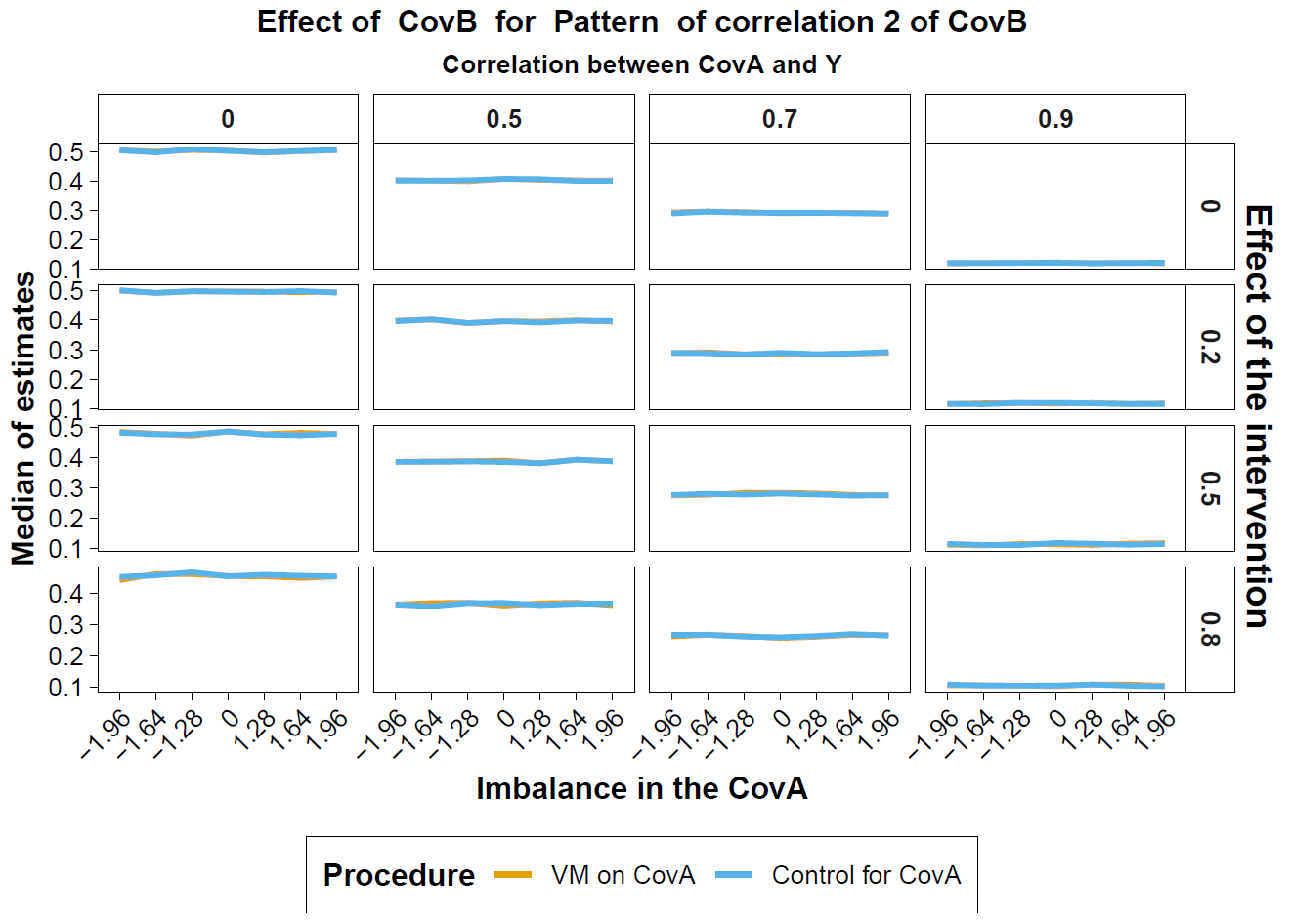


Figure S18. Median of estimates (y-axis; regression coefficients) for the effect of CovB for pattern of correlation 2 of CovB in the ANCOVA (Y ~ CovA + CovB + Group) separately for the VM procedure (orange lines) and control for CovA approach (blue lines) across imbalances of the covariate A (x-axis) when the sample size varied according to the effect size to be detected (rows; absent=0, n=788; small=0.2, n=788; medium=0.5, n=128; large=0.8, n=52) and the correlation between the covariate A and the dependent variable Y ranged between 0 and 0.9 (columns).


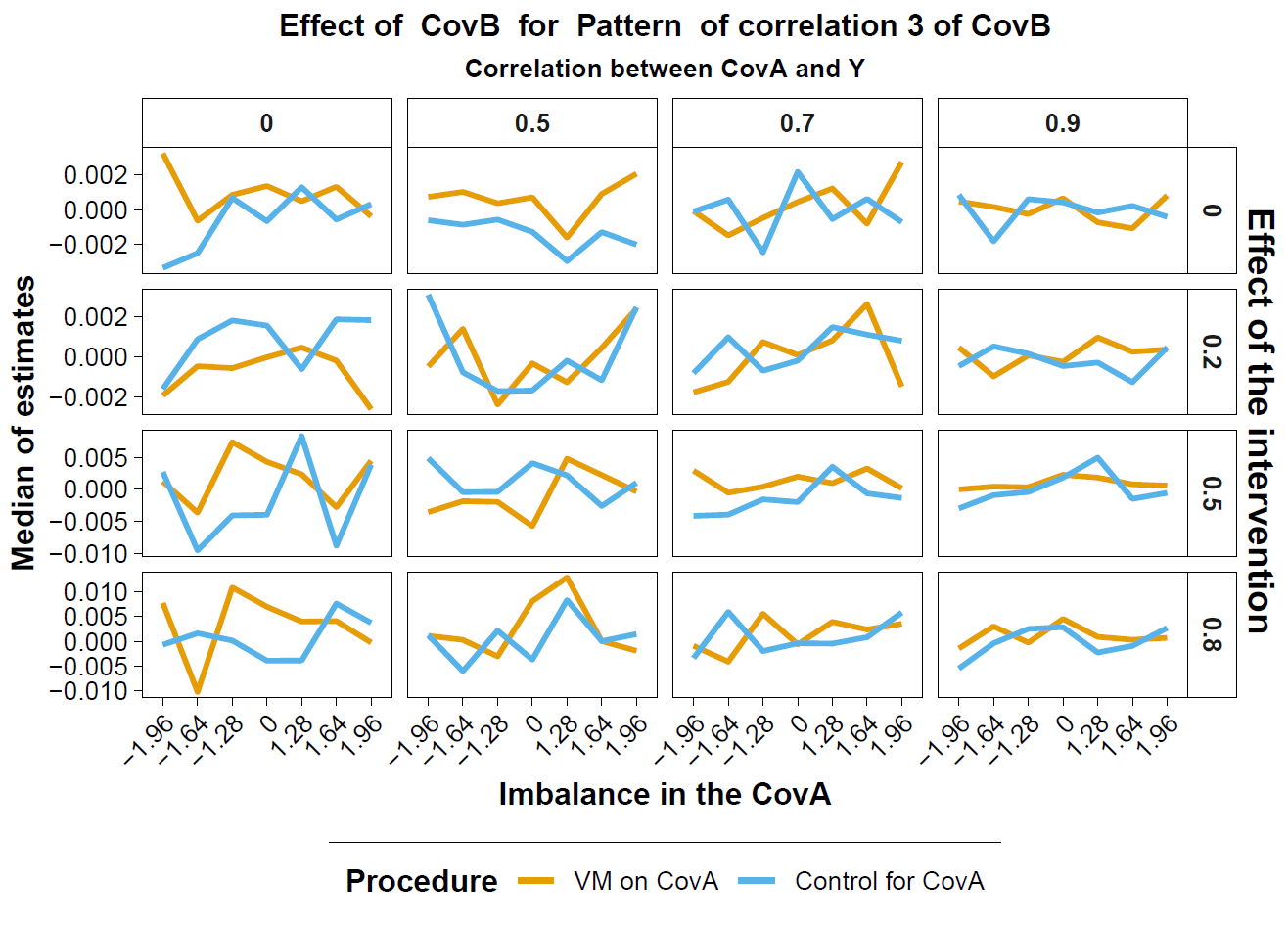


Figure S19. Median of estimates (y-axis; regression coefficients) for the effect of CovB for pattern of correlation 3 of CovB in the ANCOVA (Y ~ CovA + CovB + Group) separately for the VM procedure (orange lines) and control for CovA approach (blue lines) across imbalances of the covariate A (x-axis) when the sample size varied according to the effect size to be detected (rows; absent=0, n=788; small=0.2, n=788; medium=0.5, n=128; large=0.8, n=52) and the correlation between the covariate A and the dependent variable Y ranged between 0 and 0.9 (columns).


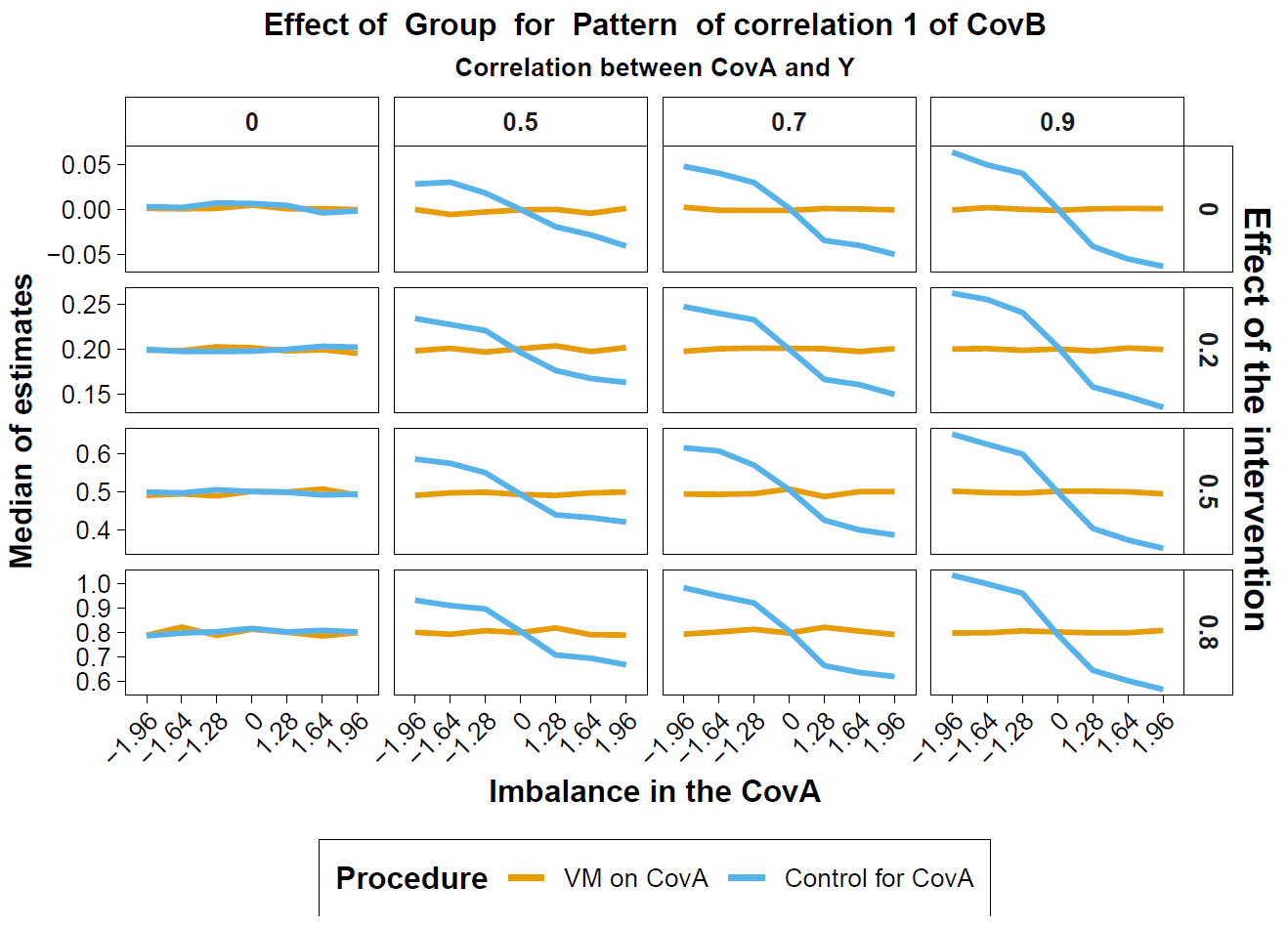


Figure S20. Median of estimates (y-axis; regression coefficients) for the effect of Group for pattern of correlation 1 of CovB in the ANCOVA (Y ~ CovA + CovB + Group) separately for the VM procedure (orange lines) and control for CovA approach (blue lines) across imbalances of the covariate A (x-axis) when the sample size varied according to the effect size to be detected (rows; absent=0, n=788; small=0.2, n=788; medium=0.5, n=128; large=0.8, n=52) and the correlation between the covariate A and the dependent variable Y ranged between 0 and 0.9 (columns).


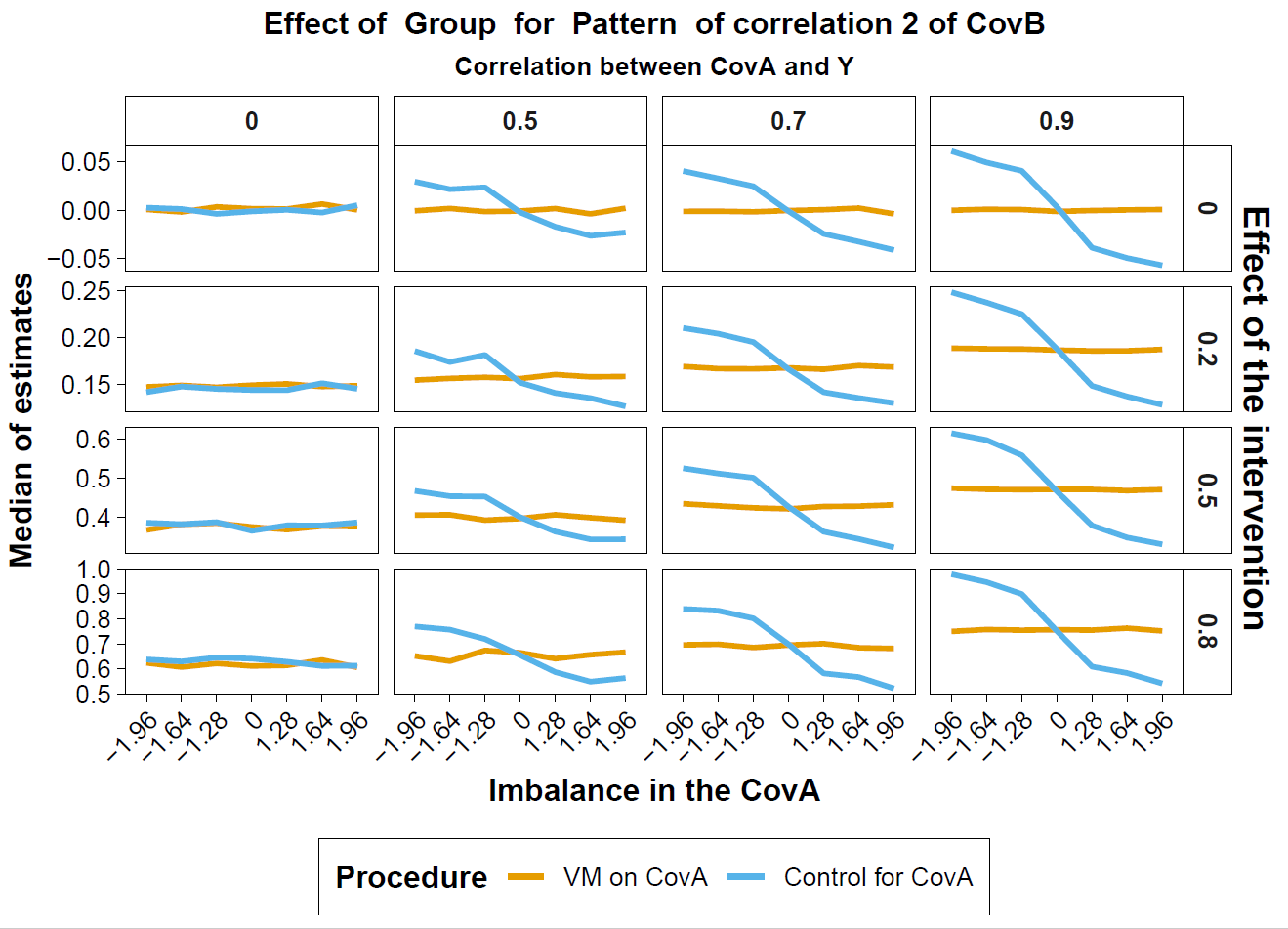


Figure S21. Median of estimates (y-axis; regression coefficients) for the effect of Group for pattern of correlation 2 of CovB in the ANCOVA (Y ~ CovA + CovB + Group) separately for the VM procedure (orange lines) and control for CovA approach (blue lines) across imbalances of the covariate A (x-axis) when the sample size varied according to the effect size to be detected (rows; absent=0, n=788; small=0.2, n=788; medium=0.5, n=128; large=0.8, n=52) and the correlation between the covariate A and the dependent variable Y ranged between 0 and 0.9 (columns).


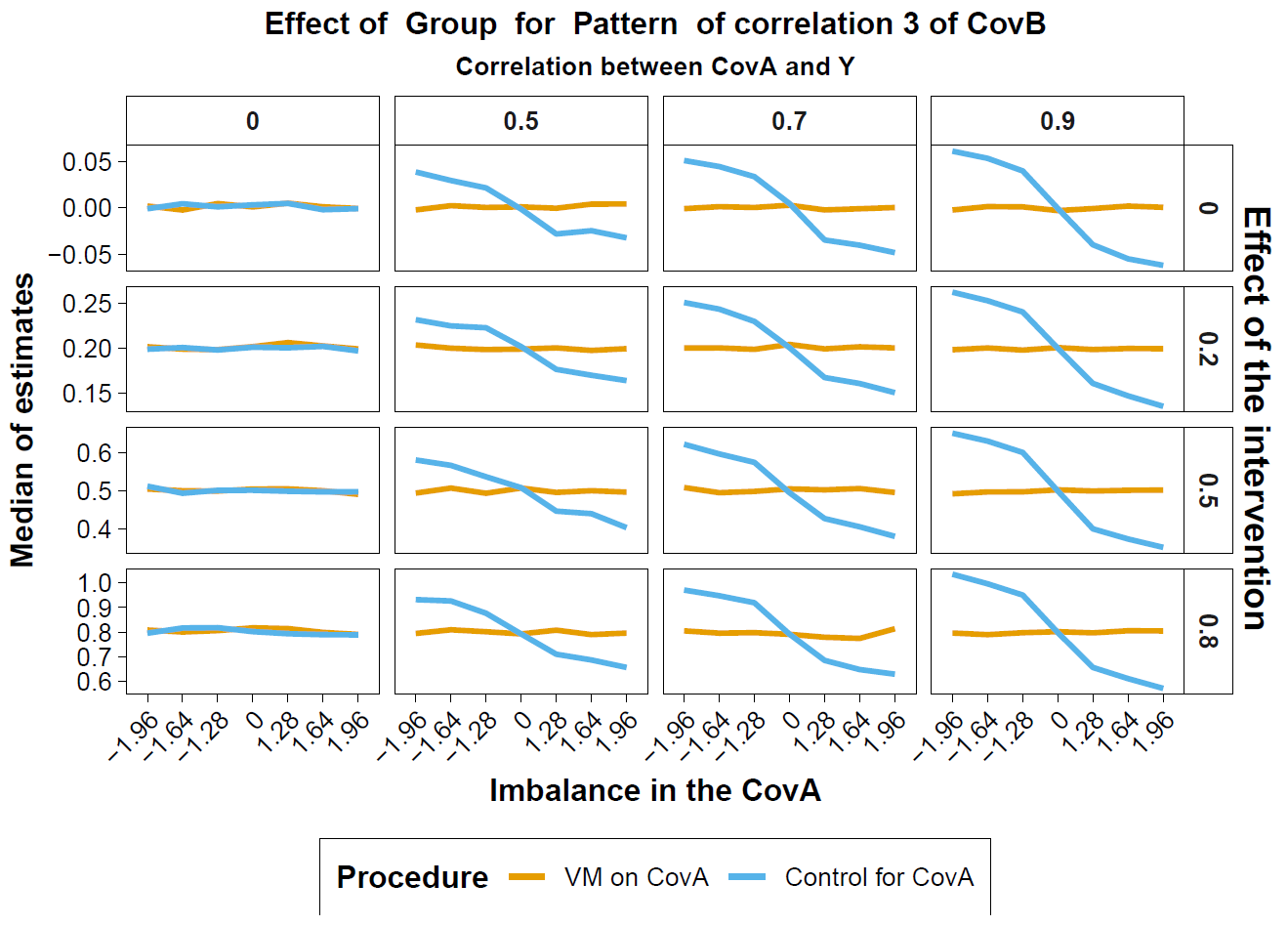


Figure S22. Median of estimates (y-axis; regression coefficients) for the effect of Group for pattern of correlation 3 of CovB in the ANCOVA (Y ~ CovA + CovB + Group) separately for the VM procedure (orange lines) and control for CovA approach (blue lines) across imbalances of the covariate A (x-axis) when the sample size varied according to the effect size to be detected (rows; absent=0, n=788; small=0.2, n=788; medium=0.5, n=128; large=0.8, n=52) and the correlation between the covariate A and the dependent variable Y ranged between 0 and 0.9 (columns).
